# Socioeconomic differences in the long-term effects of teacher absence on student outcomes

**DOI:** 10.1080/14616696.2023.2212744

**Published:** 2023-05-20

**Authors:** Nicolai Topstad Borgen, Simen Markussen, Oddbjørn Raaum

**Affiliations:** a Department of Special Needs Education, University of Oslo, Oslo, Norway; b Center for Research on Equality in Education, University of Oslo, Oslo, Norway; c Department of Sociology and Human Geography, University of Oslo, Oslo, Norway; d Centre for the Study of Professions, Oslo Metropolitan University, Oslo, Norway; e Ragnar Frisch Centre for Economic Research, Oslo, Norway

**Keywords:** Teacher absence, teacher effectiveness, dropout, academic achievement, socioeconomic gaps, compensatory advantage

## Abstract

School teachers’ sickness absence has been shown to affect student achievement in the short run. However, we know little about whether socioeconomic backgrounds may compensate for reductions in instructional quality and to what extent teacher absence effects persist over time. This paper examines the socioeconomic differences in the short- and long-term effects of teacher absence. We use population-wide Norwegian register data to study the effects of certified teacher absence during lower secondary school (grades 8–10) on non-completion of upper secondary education by age 21 (i.e. school dropout) as well as academic achievement in 10th grade. In a school fixed effects model, we find that an increase in teacher absence of 5 percentage points reduces students’ examination grades by 2.3% of a standard deviation and increases the dropout probability by 0.6 percentage points. However, the teacher absence effects vary considerably by family background, with large effects for low-SES students driving the overall effects. Overall, our findings indicate that reductions in instructional quality increase social inequality in long-term educational outcomes. This result highlights that studying heterogeneous impacts of contextual exposures is needed to understand the role of schools in shaping inequality.

## Introduction

The effects of teacher qualifications on student learning, and its implications for social inequality, have been debated since the seminal Coleman report (Coleman *et al*. 1966) and have received an increasing focus from scientific studies across different disciplines (Reimer 2019; Morgan and Shackelford 2018). Several studies have demonstrated that teacher effectiveness is highly important for student learning (Hanushek and Rivkin 2006; Hanushek and Rivkin 2010; Kelly *et al*. 2018; Lee 2018), but there is less evidence on what makes a teacher good (or bad). As teaching quality has become a top priority on the education policy agenda around the world (Darling-Hammond 2017), the presence of stable teachers is a potentially important element, and some of the variance in student learning may stem from the *absence* of teachers (Miller *et al*. 2008; Kim *et al*. 2019).

Teacher absence is hypothesized to have sizeable effects on student achievement. For example, when the regular teacher is absent, the school is likely to substitute this teacher with a less qualified instructor or cancel class altogether because of the scarcity of substitute teachers (Miller *et al*. 2008; Bowers 2001). Additionally, teacher absence may result in disruption of classroom routines, lack of meaningful student-teacher relationships, lack of knowledge about students’ specific skills, and failure to implement long-term instructional strategies (Miller *et al*. 2008). In line with this reasoning, recent studies have credibly identified adverse short-term effects of teacher absence on students’ academic achievements (Miller *et al*. 2008; Clotfelter *et al*. 2009; Herrmann and Rockoff 2012).

However, we know little about whether teacher absence has heterogeneous effects by socioeconomic background and, in particular, whether these are important for students in the long run. The literature on teacher absence is scarce, consisting only of a handful of studies examining short-term average effects in grades 4–8 in the US. On the one hand, we know that poor academic achievements in primary school are a good predictor of long-term academic failure (Falch *et al*. 2014). On the other, we know from related research fields, such as the class size literature, that there may be substantial differences in effect sizes between countries (Wößmann and West 2006) and that the short-term effects on test scores may fade out (Chetty *et al*. 2011). Whether teacher absence effects are transmitted into long-term consequences is crucial (Bailey *et al*. 2017); if they fade out, they are of little concern, as the damage created in the short term gets repaired. However, there is ground for concern if the (differential) effects of teacher absence on student outcomes are long-lasting, resulting in a loss in student learning and increasing socioeconomic inequality.

This paper contributes to the literature on teacher absence by uncovering whether teacher absence has long-term impacts and to what extent these impacts vary by students’ socioeconomic backgrounds. We use population-wide Norwegian register data to study the effects of teacher absence during lower secondary school (8–10th grade) on non-completion of upper secondary education five years later (by age 21). Further, we examine whether non-completion is explained by short-term impairment of academic achievements (as measured by exam scores at the end of grade 10). Teacher absence effects are identified using a school fixed effects model. Since students with low achievements are at high risk of school dropout, we also use quantile regression to examine whether the effects of teacher absence vary across the grade distribution. Finally, we examine whether the consequences of teacher absence are less severe for students of high socioeconomic backgrounds than for other students.

The main contribution of this paper to the social stratification literature is to illustrate how socioeconomic differences in the effects of contextual exposures may result in persistent educational inequality. The jury is still out on whether school heterogeneity reinforces or mitigates socioeconomic gradients in academic achievement and educational attainment (Downey and Condron 2016; Passaretta and Skopek 2021). While proponents of the reinforcement view point to unequal distribution of school resources along the lines of socioeconomic segregation in the US context (Adamson and Darling-Hammond 2012), compensating allocation of resources where schools with disadvantaged students have a lower student-teacher ratio is more prominent in other countries (Wößmann and West 2006). However, as we demonstrate in this paper, even if school resources are equal across socioeconomic groups, the effect of low school quality may differ, thereby contributing to educational inequality (Raudenbush and Eschmann 2015). The mechanisms responsible for socioeconomic gradients in teacher absence effects are likely similar to those at play when instructional quality drops, as during school closures due to the COVID-19 pandemic (Engzell *et al*. 2021). As such, this paper concerns the interactions between instructional quality deficiencies and family background more generally.

## Teacher absence and socioeconomic gradients

### Effects of teacher absence

The rate of teacher absence is about 5–6% in countries such as the United States (Herrmann and Rockoff 2012) and Norway (Rønning 2012), and about 3% in the United Kingdom and Australia (Bradley *et al*. 2007).^1^ These rates amount to about 6–9 days per school year for an average teacher in these countries. In the US, absence rates are highest at schools that serve low-income students (Clotfelter *et al*. 2009).

There are two main reasons why teacher absences may impair student achievement. The first mechanism relates to an inferior teacher substitute. Teacher quality is among the most important school factors influencing student outcomes (Darling-Hammond 2000; Chetty *et al*. 2014). A shortage of qualified substitute teachers means that absent teachers are often replaced with unqualified teachers, and sometimes classes are simply canceled because of the scarcity of substitute teachers (Sutcher *et al*. 2019). Thus, the absence of teachers is likely to lead to a radical drop in instructional quality (Miller *et al*. 2008).

A second mechanism operates via a disruptive environment. Even if the teaching of the substitute holds a high level, the absence of the regular teacher will disrupt classroom routines, and the substitute will likely struggle to implement the regular teacher’s long-term instructional strategies (Miller *et al*. 2008). Moreover, differentiated instruction that meets the student’s individual needs builds upon knowledge about students’ strengths and challenges (Dixon *et al*. 2014), and substitutes may lack this knowledge about students’ skill levels. Further, especially for students struggling in school, positive student-teacher relationships may be a protective force, with teachers serving as mentors and sources of support (Crosnoe *et al*. 2004; Davis 2003). However, it may be challenging for students to form meaningful relationships with multiple substitutes (Miller *et al*. 2008). Finally, teacher absence may have a disruptive organizational influence on the school as a whole (Ronfeldt *et al*. 2013). Besides affecting workplace morale (Rønning 2012), teacher absence consumes administrative resources in finding and paying substitute teachers (Clotfelter *et al*. 2009), resources that could instead be used on improving teaching and working conditions (Ronfeldt *et al*. 2013).

Only a few studies have credibly investigated whether teacher absence impairs student learning. Identifying a causal effect requires an empirical strategy that accounts for three sources of bias, all of which are likely to exaggerate the true effect. First, there is a potential negative correlation between teacher absence and teacher qualifications (Miller *et al*. 2008). Second, there is a possible reverse causality issue if the level of behavioral problems among the students affects teacher absence, and students with behavioral problems struggle academically (Clotfelter *et al*. 2009). Third, common shocks – such as an influenza outbreak – may increase teacher absence and directly affect students’ learning.

Three studies we know of have used a design that credibly accounts for these potential biases, all of which find that teacher absence harms short-term student outcomes (Miller *et al*. 2008; Clotfelter *et al*. 2009; Herrmann and Rockoff 2012).^2^ Miller *et al*. (2008) have access to student-teacher-year data and include teacher fixed effects in a value-added model to account for all time-invariant teacher characteristics. They find that teacher absence impairs students’ achievements, suggesting that ten additional absence days reduce fourth-grade mathematics achievement by 3.2% of a standard deviation.^3^ Clotfelter *et al*. (2009), also using a value-added model with teacher fixed effects, find adverse effects of teacher absence in grades 4–5, but of a smaller magnitude than Miller *et al*. (2008). Having a teacher with ten additional absence days reduced math scores by about 1.7% of a standard deviation and reading by about 0.9% of a standard deviation. Finally, like Miller *et al*. (2008) and Clotfelter *et al*. (2009), Herrmann and Rockoff (2012) find that teacher absences reduce the achievements of students, using a value-added teacher fixed effects model. They found that ten days of absence reduced students’ math and English scores in grades 4–8 by 1.2 and 0.6% of a standard deviation, respectively.

### Socioeconomic gradients

A student’s academic development is influenced jointly by the quality of the teaching and her childhood environment. It is likely that a reduction in instructional quality – such as teacher absence – induces a compensatory response from parents in terms of shifts in household resources toward education (Becker and Tomes 1976; Todd and Wolpin 2003; Bonesrønning 2004; Houtenville and Conway 2008). Further, this compensatory response may vary with socioeconomic background. High-resource families often compensate for any early disadvantage, as shown in studies of birthweight effects (Torche and Echevarría 2011), sibship size effects (Tanskanen *et al*. 2016), and month of birth effects (Bernardi and Grätz 2015).

Disadvantageous events such as teacher absence may be less harmful to children of high SES parents because they have resources to buy services such as tutoring (Tanskanen *et al*. 2016) or simply instruct their kids more effectively. Another reason is that middle-class parents have higher educational expectations for their children (Roksa and Potter 2011) and may, to a greater extent, deliberatively cultivate their children for academic success (Lareau 2011). Parental involvement with school and children’s academic development differs by socioeconomic background (Lareau 2011). Unlike middle-class parents, working-class parents give schools the primary responsibility for developing their children’s cognitive skills. Consequently, teacher absence may be less likely to prompt compensatory responses among parents with low education.^4^

The effects of teacher absence may be stronger for disadvantaged children for other reasons than a weak compensatory response from parents. If school and family inputs are substitutes, diminishing returns to inputs imply that school quality variation has less impact on students of high socioeconomic backgrounds. Disadvantaged children may suffer from less developmental stimulation from parents, and their human capital accumulation responds more strongly to changes in the learning environment in kindergarten and school. Such differential sensitivity to instructional quality by socioeconomic background is consistent with evidence that school and preschool programs are particularly important for disadvantaged children (Borman and Kimball 2005; Havnes and Mogstad 2015; Zachrisson *et al*. 2023). Additionally, differences by socioeconomic background in non-cognitive skills (Anger and Schnitzlein 2017) and the degree of behavioral problems (Mcleod and Kaiser 2004) may contribute to disadvantaged students typically being more sensitive to disruptions in the learning environment. Studies have also suggested that a good student-teacher match serves as a protective force, especially for disadvantaged students and students at risk of academic failure (Crosnoe *et al*. 2004; Muller 2001). Teacher absence that disrupts such favorable relationships may be particularly harmful to these vulnerable children.

In line with these latter arguments, Clotfelter *et al*. (2009) find that short-term grades of disadvantaged students are most hurt by teacher absence. However, we do not know whether these short-term differential effects persist into adulthood.

## Data and variables

### Register data and analysis sample

We use longitudinal population-wide Norwegian register data covering students born 1986–1999 who attend lower secondary schools (grades 8–10), excluding children of immigrants who arrive in Norway after school start (6 years old). Compulsory education in Norway starts at the age of six and lasts for ten years, with primary education in grades 1–7 (ages 6–13) and lower secondary education in grades 8–10 (ages 13–16). Few students receive compulsory education in private schools (about 4%), and all schools are publicly funded. Both private and public schools are included in our data. There are three main types of schools in Norway: elementary schools (grades 1–7), lower secondary schools (grades 8–10), and combined elementary and lower secondary schools (grades 1–10). About 60% of the schools that provide lower secondary education are combined compulsory and lower secondary schools, and only 40% are lower secondary schools only. However, lower secondary schools are larger in terms of enrollment; the 40% lower secondary schools contain 74% of our study’s birth cohorts.

After completing lower secondary education, more than nine in ten students enter upper secondary education. Students can choose between multiple vocational and academic study tracks; the academic tracks take three years and result in higher education qualification, while vocational usually last four years, with two years in school followed by a two-year apprenticeship.

### Teacher absence variable

Our exposure variable measures teacher’s sickness absence at the school level. Measuring the exposure at the overall school level is typical in various studies of school factors, such as studies of school resources (Jackson and Mackevicius 2021), school quality (Angrist *et al*. 2017), and student composition (Hermansen and Birkelund 2015). Further, a school-level measure has the advantage of capturing externalities for students not directly influenced by the absence, caused by the absences’ disruptive organizational influence on the school as a whole. Still, ideally, we would also like to track daily absences for all teachers and be able to link teachers to all students they have instructed throughout their school careers. The perfect teacher absence exposure for each individual student would have reflected the time each student was planned to spend with their teacher. Unfortunately, however, such data are rarely available for research. Therefore, this paper’s teacher absence measure is the average of the long-term teacher absence rate (lost days divided by the total number of days) certified by a doctor, aggregated to the school level during the three years when the student attended 8th, 9th, and 10th grade. This section discusses what we can learn from using this teacher absence measure.

Absence records stem from individual teacher records in the historical event database (FD-Trygd), which includes all sickness absence spells lasting 16 days or more. All such spells are covered by the social security system in Norway (from day 17), and a physician must certify them. The replacement rate is 100% during the absence, up to a ceiling amounting to 6G or around 60,000 USD in annual earnings. The employer covers the first 16 days of absence, while the social security system covers the rest (up to one year). Absence information from administrative registers is more accurate than self-reported sickness absences (e.g. Kristensen *et al*. 2018; Short *et al*. 2009), partly because the reimbursement generates an incentive for employers to accurately report absences (Kristensen *et al*. 2018; Markussen *et al*. 2012). However, our absence measure from the register data excludes other reasons for absence, such as funerals or conferences. Moreover, it excludes certified sickness absences of fewer than 16 days and self-certified sick leave, which may be more detrimental than long-term absences (Herrmann and Rockoff 2012). For a few cohorts, we can observe all certified sickness absences: the correlation between long-term certified sickness absences and all certified sickness absences is 0.92, suggesting that results would be fairly similar had we observed all sickness absences. Nevertheless, if the (unobserved) shorter absence spells are correlated with longer-term absences, we may slightly overestimate the effects of long-term absences. The Supplementary Online Appendix D discusses this potential bias in more detail, suggesting that our model may overestimate the effects of long-term absence by about 11%.

The teacher-student matching is based on a unique school identifier that allows us to match students (from the National Education Database) and teachers (from the Register of Employers and Employees) to schools in Norway. We cannot match individual students and teachers within schools, but the teacher absence spells can be dated as part of a specific school year. For each school year, we observe the average long-term teacher absence rate (lost days divided by the total number of days) certified by a doctor. Our treatment variable is the teacher absence at the student level (exposure), defined as the school average during the three school years when the student attended 8th, 9th, and 10th grade. Aggregating teacher absence at the school level results in an error-ridden measure of individual-level teacher absence exposure. Nevertheless, in the following paragraphs, we argue that this aggregation leads to less precision but *not* the attenuation bias one might expect. Moreover, the absence among all teachers also captures the indirect effects through disruptive organizational influences, which an individual student-teacher absence exposure would fail to include.

First, we aggregate teacher absence across different classes within the same grade. Unlike with classical measurement error, where the measurement error is uncorrelated with the true treatment variable, this type of measurement error is uncorrelated with the observed (aggregated) treatment variable, sometimes known as optimal prediction error (Hyslop and Imbens 2001). Importantly, this type of measurement error results in consistent point estimates, and the main drawback of aggregating teacher absence at the school cohort level is larger standard errors.

Second, we aggregate teacher absence across different grades within the same school. Obviously, this aggregation results in an error-ridden measure of students’ exposure to teacher absence. However, this aggregation is another example of optimal predictors that results in consistent but less precise estimates, as the measurement error is by definition independent of the observed value (Hyslop and Imbens 2001). To fix ideas, consider a model where the outcome ($Y_{\mathrm{isc}}$) of student i of cohort c in school s depends only on individual-level exposure to teacher absence during each of the three school years ($T_{\mathrm{scg}},\;\;g=8,\;\;9,\;\;\;\mathrm{and}\;10$):

$$Y_{\mathrm{isc}}=a+\overset{10}{\underset{g=8}{\sum }}\beta_{g}T_{\mathrm{scg}}+\varepsilon_{\mathrm{isc}}$$

We do not observe teacher absence at the grade level but instead at the school level. For example, for cohort c in their final year, we observe the school-level average $TS_{\mathrm{sc}10}=(T_{s(c+2)8}+T_{s(c+1)9}+T_{\mathrm{sc}10})/3$. For simplicity, let us say that (i) only teacher absence in grade 10 matters ($\beta_{8}=\beta_{9}=0$) and (ii) that the absence rates of these three groups of teachers have the same variance ($\sigma_{g}^{2}$). Then, the two biases are of equal magnitude, operate in opposite directions, and cancel out, as explained in the following paragraph and illustrated in the data simulations in Appendix C.

First, the measurement error results in a downward bias in the covariance between measured teacher absence ($TS_{\mathrm{sc}10}$) and the outcome, since only one-third of the variation in $TS_{\mathrm{sc}10}$ is relevant for the outcome. Simultaneously, taking the average of absence rates across 8th, 9th, and 10th-grade teachers scales down the variance in the measured teacher absence variable by an equal amount (compared to the true variance among tenth-grade teachers), which exactly cancels out any influence of the measurement error on the covariance. Thus, replacing the true tenth-grade teacher absence ($T_{\mathrm{sc}10}$) with the error-ridden observed school-level teacher absence ($TS_{\mathrm{sc}10}$) produce consistent estimates of the true effect of teacher absence in tenth grade, only less precisely estimated than if we could directly observe the absence of 10th-grade teachers.

This argument extends to a model where teacher absence during grades 8 and 9 also matters, and we measure the school average over all three grades: $TS_{\mathrm{sc}}=\frac{TS_{\mathrm{sc}8}+TS_{\mathrm{sc}9}+TS_{\mathrm{sc}10}}{3}$. Thus, the interpretation of our estimated parameter is the effect on student outcomes by increasing teacher absence in all three years of lower secondary school. Appendix C uses a data simulation to show that replacing the true teacher absence with the error-ridden observed school-level teacher absence variable produces unbiased estimates of the teacher absence effect. In sum, our teacher absence measured at the school level captures the direct impact for the student involved (e.g. lower quality of substitutes) as well as indirect influences through a disrupted organization (e.g. less effective teacher teamwork).

### Outcomes

Long-term effects on educational attainment are measured by non-completion of upper secondary education at age 21 (labeled dropout). By extending the study beyond test (or exam) scores, we add to the literature by looking at long-term effects on an outcome important to individual lifetime welfare. School dropout is definitely a high stake and associated with substantially reduced lifetime earnings. An illustration can be taken from the adult five-year average earnings distribution, where the differential between those who complete and those who do not are more than 12 percentiles (Online Appendix Table A6). More education is also likely to buy more than labor market success (Oreopoulos and Salvanes 2011), such as fewer health problems (e.g. Hoff *et al*. 2018). Following previous studies, we also investigate the effects of teacher absence on short-term academic performance, defined as the grade obtained in the final examination in lower secondary school (10th grade). Final exams consist of written and oral tests, with the written for the cohorts included in our study being math, Norwegian, and foreign language (English), and the oral being math, Norwegian, foreign language (English), religion and ethics, science and environmental, and social science. Students are randomly drawn to different tests through an exam lottery, and typically take 2–3 exams. These final exams are taken at the end of grade 10 and are externally and anonymously graded.

### Socioeconomic background and control variables

Individual control variables are graduation cohort (dummies), gender, average of father’s earnings while offspring student aged 11–15 (linear and quadratic term), average of mother’s earnings at age 11–15 (linear and quadratic term), father’s educational level (9 dummies), mother’s educational level (9 dummies), immigrant background (dummies), age of immigration, whether father received social welfare at age 10–12, whether mother received social welfare at age 10–12, whether father received criminal charge at age 10–12, whether mother received criminal charge at age 10–12, birth order (linear and quadratic term), number of siblings (linear and quadratic term), father’s age at birth (linear and quadratic term), and mothers’ age of birth (linear and quadratic term).

At the school level, we control for the female share of teachers, the average age of teachers, the share of teachers with a master’s degree, the share of teachers with higher education within teacher training and pedagogy (based on The Norwegian Standard Classification of Education), and the immigrant share among the teachers.

## Methods

### School fixed effects model

Several school, teacher, and individual characteristics are likely correlated with teacher absence *and* student outcomes. For example, in Norway, principals hire teachers from the pool of applicants, and popular schools may be able to hire better teachers, which may also be less absent. Unless properly accounted for, teacher absence effect estimates are likely biased. We apply a school fixed effects model with controls for observable student and teacher characteristics:

(1) $$Y_{\mathrm{isc}}=\alpha +\mathrm{\delta T}S_{\mathrm{sc}}+\theta_{1}X_{\mathrm{isc}}+\theta_{2}M_{\mathrm{sc}}+\eta_{s}+\gamma_{c}+\varepsilon_{\mathrm{isc}},\;$$

where i, s, and c index individual, school, and cohort, respectively. Y is the outcome variable, TS is the average teacher absence rate measured at the school-cohort level during grades 8–10, X is individual control variables, and M is teacher characteristics for each school cohort. The school fixed effect, $\eta_{s}$, captures all time-invariant differences between schools, such as stable teacher characteristics, student characteristics, and school resources. Within-school changes in student and teacher characteristics, such as an increase in students with behavioral problems, are adjusted for by means of control variables. Finally, the cohort fixed effect $\gamma_{c}$ captures all mean differences between students across graduation years.

Our main parameter of interest is δ, the regression coefficient for teacher absence on student performance. To give δ a causal interpretation, there must be no unobserved factors at the student *or* teacher level that correlate with teacher absence and influence student outcomes. The first concern is that there may be a negative correlation between teacher absence and teacher qualifications/school management effectiveness. For example, high-quality teachers may lead to less disturbance and a better learning environment for the students, resulting in less teacher absence. Having a high absence rate may also suggest that a teacher lacks skill or effort when teaching (Miller *et al*. 2008). Clotfelter *et al*. (2009) and Herrmann and Rockoff (2012) find that including teacher fixed effects shrinks the estimated effect of teacher absence. However, the evidence on quality-absence correlation is inconclusive; Miller *et al*. (2008) suggest that teachers with weak (unobserved) teaching skills are not more likely to be absent. In our school fixed effects model, we control for stable teacher and organizational characteristics at the school by means of school fixed effects and account for observable teacher characteristics that may change within schools (e.g. teachers’ educational level).

Second, there may be a correlation between student characteristics and teacher absence. Studies have found that schools that serve disadvantaged children have persistently higher absence rates (Clotfelter *et al*. 2009). Because of the association between academic failure and behavioral problems (Evensen *et al*. 2016; Wertz *et al*. 2018), we may expect that students who struggle academically cause teachers to get sick leaves (reverse causality). This absence may, in turn, affect the performance of these vulnerable students (simultaneity bias). In our model, some of these factors are accounted for by including school fixed effects, and we account for family background by including variables such as parental education, parental earnings, parental social welfare, and parental criminal records. We also perform a series of robustness checks, which all indicate that the identifying assumptions are not violated (discussed in depth in the result section below).

### Quantile treatment effects

The main analyses use ordinary least squares (OLS) to examine whether teacher absence affects long-term school dropout and short-term test scores (exams). To shed light on the role of short-term effects in explaining heterogeneous effects on school dropout, we also study how the short-term effects of teacher absence vary by students’ achievement level, using quantile regressions. Whether teacher absence translates into long-term impacts on dropout depends on whether teacher absence reduces the achievement level of high-achieving or low-achieving students. Suppose teacher absence depresses the academic achievements of high-achievers only. In that case, any impact on dropout is likely to be small simply because nearly all the affected students are completing upper secondary school anyway. In contrast, if teacher absence depresses low achievers’ academic achievement, then any effect on dropout is potentially large because many of them are at risk.

To get a complete view of how teacher absence affects academic achievements, we identify unconditional quantile treatment effects (QTE) using the residualized quantile regression (RQR) model (Borgen *et al*. 2021b; Borgen *et al*. 2021a).^5^ Assuming rank invariance, the RQR model allows for examining whether students who have low achievement levels are affected differently by teacher absence than students with high achievement levels. Examination grades are calculated as the average test score of 2–3 examination grades, resulting in a discrete outcome distribution. We solve this problem by artificially smoothing the data using the jittering approach suggested by Machado and Silva (2005), which is similar in spirit to the over-smoothing approach Firpo *et al*. (2009) suggest in the RIF-OLS model. Specifically, we add uniform noise to jitter the outcome, using a uniform distribution over the interval [−0.5,0.5]. To test whether the results are sensitive to this artificial smoothing, we compare effects on smoothed examination grades with effects on grade point average. The grade point average, which consists of teacher-assigned and externally graded exams, is more prone to grading on a curve; however, it should capture much of the same teacher absence effect. There is no need to smooth the grade point average variable since it consists of eleven subjects. Using the (unsmoothed) grade point average produces similar results as the smoothed examination grades (Appendix Figure A5).

## Results

### Descriptive statistics

Our data’s average certified teacher absence rate is about 4.7%, which amounts to between eight and nine days of long-term absence per teacher during each school year (Table 1). This absence does not include short-term certified (<17 days) or self-certified absences. Only 10% of the students are exposed to a teacher absence rate of less than 2.2%, and only 10% are exposed to a rate of more than 7.5% (Appendix Table A1, Appendix Figure A1 and A2). Thus, a difference of about ten absence days (i.e. 5.3 percentage points) separates the 90th and 10th percentiles. Some schools have persistently higher absence rates than others (Appendix Table A10), resulting in a correlation between students’ exposure to school-level teacher absence across grades of about 0.22 ($\rho_{8\mathrm{th},9\mathrm{th}}$), 0.16 ($\rho_{8\mathrm{th},10\mathrm{th}}$), and 0.24 ($\rho_{9\mathrm{th},10\mathrm{th}}$) (Appendix Table A2). Nevertheless, these correlations suggest substantial variation in teacher absence across graduation cohorts within the same school.

**Table 1.** Descriptive statistics.

	*N*	Mean	SD	Min	Max
*Outcome variables*					
Examination grades (10th grade)	562,246	0.030	0.995	−3.652	2.246
Dropout (by age 21)	368,785	0.286	0.452	0	1
*Treatment variable*					
Teacher absence rate (8–10th grade)	555,155	0.047	0.025	0	0.449
*Individual controls*					
Graduation year	587,152	2008.7	3.998	2002	2015
Girl	587,152	0.486	0.500	0	1
Father’s earnings	582,368	531688.639	323459.187	0	2,722,738
Mother’s earnings	587,037	317706.540	198506.856	0	2,722,738
Father’s education					
No education	582,035	0.002	0.042	0	1
Primary education	582,035	0.003	0.054	0	1
Lower sec. education	582,035	0.198	0.399	0	1
Some upper sec. education	582,035	0.104	0.305	0	1
Upper sec. education	582,035	0.325	0.468	0	1
Post-sec. not higher education	582,035	0.053	0.223	0	1
Undergraduate education	582,035	0.205	0.403	0	1
Graduate education	582,035	0.091	0.288	0	1
Postgraduate education	582,035	0.012	0.108	0	1
Unknown	582,035	0.008	0.087	0	1
Mother’s education					
No education	586,983	0.003	0.051	0	1
Primary education	586,983	0.004	0.062	0	1
Lower sec. education	586,983	0.216	0.412	0	1
Some upper sec. education	586,983	0.114	0.317	0	1
Upper sec. education	586,983	0.260	0.439	0	1
Post-sec. not higher education	586,983	0.029	0.167	0	1
Undergraduate education	586,983	0.311	0.463	0	1
Graduate education	586,983	0.053	0.224	0	1
Postgraduate education	586,983	0.006	0.074	0	1
Unknown	586,983	0.005	0.073	0	1
Country of birth					
NOR-born to NOR-born parents	587,147	0.882	0.322	0	1
FOR-born with two FOR-born parents	587,147	0.005	0.071	0	1
NOR-born to FOR-born parents	587,147	0.038	0.191	0	1
FOR-born with one NOR-born	587,147	0.001	0.038	0	1
NOR-born with one FOR-born parent	587,147	0.072	0.258	0	1
FOR-born to NOR-born parents	587,147	0.002	0.041	0	1
Year of immigration	587,152	0.028	0.367	0	6
Father social welfare	586,659	0.054	0.226	0	1
Father criminal charge	586,659	0.028	0.165	0	1
Mother criminal charge	586,659	0.007	0.084	0	1
Mother social welfare	586,659	0.062	0.241	0	1
Sibling order	587,150	1.887	0.988	0	17
Number of siblings	587,133	1.973	1.232	0	18
Father’s age	582,394	31.402	5.837	10	75
Mother’s age	587,043	28.544	5.016	13	54
*Teacher controls*					
Teacher female	562,843	0.599	0.105	0	1
Teacher age	562,843	46.180	3.341	24.5	70
Teacher immigrant background	562,843	0.083	0.068	0	1
Teacher graduate education	562,843	0.116	0.087	0	1
Education within teacher training and pedagogy	562,843	0.724	0.132	0	1

Note: NOR = Norwegian, FOR = Foreign.

Teacher absence is weakly related to the students’ socioeconomic and immigrant background (Appendix Table A7, Appendix Figure A4). For example, merely 0.5 percentage points of teacher absence separate students of the parents in the lower half of the earnings distribution from the parents in the top 1%.

### Average effects on test scores and dropout

The main results are presented in Table 2. We find that teacher absence during lower secondary school significantly reduces examination grades in 10th grade. An increase in teacher absence rate of five percentage points – roughly equivalent to ten absence days or a comparison of a student at the 90th percentile of the teacher absence distribution with a student at the 10th percentile – reduces examination grades on average by 2.3% of a standard deviation (column 3). Teacher absence also significantly affects school dropout five years later. We find that a five percentage points increase in teacher absence increases dropout by 0.6 percentage points (column 6), corresponding to a 2.1% increase in the dropout rate (teacher absence effect divided by the overall dropout rate of 28.6%). Table 2 also reveals that estimates of teacher absence effects are upward biased unless we control for time-invariant differences across schools by means of school fixed effects (compare columns 2 and 5 with 3 and 6).

**Table 2.** The estimated effects of teacher absence without any control variables (columns 1 and 4), with observed individual and teacher characteristics (columns 2 and 5), and with the full fixed effects model (columns 3 and 6).

	(1)	(2)	(3)	(4)	(5)	(6)
	Examination grades	Examination grades	Examination grades	Dropout	Dropout	Dropout
Teacher absence	−0.8389**	−0.6772***	−0.4612**	0.1725*	0.1926***	0.1109**
	(0.2708)	(0.1470)	(0.1430)	(0.0874)	(0.0408)	(0.0361)
Control variables		Yes	Yes		Yes	Yes
Cohort FE		Yes	Yes		Yes	Yes
School FE			Yes			Yes
Observations	539,036	534,078	534,078	349,463	346,551	346,551

Note: Standard errors clustered at schools in parentheses.

*p < 0.05, **p < 0.01, ***p < 0.001.

### Heterogeneous effects of teacher absence

**Figure 1.**
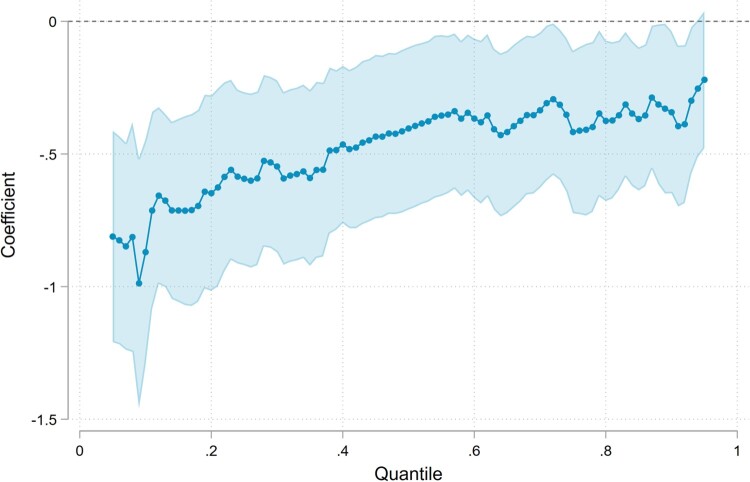
Effects of teacher absence on examination grades using quantile regressions. Note: Appendix Table A4 includes point estimates and standard errors of the estimated effect as well as tests of significance of effects across quantiles. The shaded areas show 95% confidence intervals based on 500 cluster-bootstrapped standard errors.

We find that the effects of teacher absence vary considerably by students’ achievement level and family background. Using quantile regressions, we find that the effect of teacher absence is more harmful to low-performing students (Figure 1). While teacher absence depresses examination grades across the entire outcome distribution, the effects are almost three times as large at the 10th percentile compared to the 90th percentile.^6^ Specifically, a five percentage point increase in teacher absence reduces examination grades by about 4.3% among the low-achievers (10th percentile), while only about 1.7% for the high-achievers (90th percentile).

In line with the heterogeneous effects across the exam score distribution, teacher absence is considerably more detrimental for students of low socioeconomic backgrounds (Table 3). In Panel A, teacher absence effects are different for students with high- or low-income parents, defined as the parents´ rank in the earnings distribution. Column (1) and (3) reports the estimated coefficients, whereas the regression coefficients are displayed as group-specific effects in column (2) and (4). With students of low-rank parents as a reference, teacher absence effects are consistently lower and close to zero for advantaged students with parents in the upper half of the earnings distribution. Teacher absence effects by parental education point in the same direction (Panel B), but the estimates are less precise. While the interaction terms consistently show that effects are smaller for students with highly educated parents, only the higher education interaction is significant (and negative) for dropout. For example, for students of low parental education, an increase of teacher absence of 5 percentage points increases the likelihood of dropout by 1.1 percentage points (.2219*.05), compared to a 0.2 percentage point increase for high parental education students.

**Table 3.** Teacher absence effect by parental earnings and education.

	(1)	(2)	(3)	(4)
	Examination grades		School dropout	
*Panel A:*				
Teacher absence	−0.7157***		0.1943**	
	(0.1832)		(0.0721)	
Parental earnings rank				
* 1–24th Percentile	Ref.	−0.7157***	Ref.	0.1943**
		(0.1832)		(0.0721)
* 25–49th Percentile	0.0859	−0.6297***	0.0308	0.2251**
	(0.1628)	(0.1677)	(0.0867)	(0.0629)
* 50–74th Percentile	0.3036	−0.4121*	−0.0863	0.1080
	(0.1658)	(0.1618)	(0.0882)	(0.0577)
* 75–99th Percentile	0.5686***	−0.1471	−0.2463**	−0.0521
	(0.1654)	(0.1617)	(0.0861)	(0.0486)
				
Observations	533,624		346,272	
*Panel B:*				
Teacher absence	−0.6757***		0.2219**	
	(0.1626)		(0.0765)	
Parental education				
* Lower sec. educ. or lower	Ref.	−0.6757***	Ref.	0.2219**
		(0.1626)		(0.0765)
* Upper sec. educ.	0.1891	−0.4866**	−0.0859	0.1360**
	(0.1552)	(0.1515)	(0.0840)	(0.0520)
* Tertiary education	0.2984	−0.3773*	−0.1691*	0.0528
	(0.1727)	0.1761	(0.0841)	(0.0474)
				
Observations	533,624		346,272	
				
*Panel C:*				
Teacher absence	−0.6831***		0.2219***	
	(0.1487)		(0.0538)	
Parental earnings rank				
* 1–49th Percentile	Ref.	−0.6831***	Ref.	0.2219***
		(0.1487)		(0.0538)
* 50–99th Percentile	0.3732**	−0.3098*	−0.1653*	0.0566
	(0.1365)	(0.1543)	(0.0671)	(0.0608)
Parental education				
* No Tertiary education	Ref.	−0.6831***	Ref.	0.2219***
		(0.1487)		(0.0538)
* Tertiary education	0.0502	−0.6329**	−0.0498	0.1721*
	(0.1455)	(0.2100)	(0.0667)	(0.0726)
Observations	533,624		346,272	

Note: Standard errors clustered at school level in parentheses. All models with school fixed effects, cohort fixed effects, individual controls, and teacher controls. Estimates in panels are from different models. Parental earnings rank is defined as the percentile rank of children based on parental earnings relative to children in the same graduation cohort, including those with zero earnings. Parental education refers to the highest achievement of the two parents. Estimates and standard errors in columns (2) and (4) show the estimated group-specific effects of teacher absence based on (1) and (3) (computed using lincom in Stata 16.0). In Panel C, the group-specific teacher absence effect for parental earnings rank (parental education) is computed holding parental education (parental earnings rank) at no tertiary education (1–49th percentile).

*p < 0.05, **p < 0.01, ***p < 0.001.

### Robustness

When concluding on cause-and-effect relationships, it is crucial to account for factors that may confound the estimates. Based on the school fixed effects model, we find that teacher absence impairs students’ academic achievements and subsequently increases school dropout risk. Taking account of time-invariant school characteristics (i.e. school fixed effects) is seemingly important as the estimated effects of teacher absence without these fixed effects are considerably larger (Table 2). Still, a concern is that the within-school variation in teacher absence could be (partly) caused by variation in the share of students with behavioral and academic problems, even if we condition on time-variant student family background characteristics. Supplementary Online Appendix B investigates this concern empirically by exploiting data on students’ entry test scores, students’ behavioral problems (criminal charges and poor school behavior), school environment, and health visits. These supplementary analyses support our identification strategy.

### Does short-term academic achievement mediate long-term effects?

A question remains whether the impact on short-term academic achievement explains the long-term dropout effect. The exact amount of mediation is challenging to assess empirically for several reasons (VanderWeele 2015). First, while we can convincingly address the confounding of teacher absence, credible evidence on the causal association between examination grades (the mediator) and school dropout (the outcome) is hard to establish. We estimate the association between exam scores and dropout among same-sex twins to get closer to the causal effect (Appendix Table A8), interpreting the within-twin pair estimate as an upper bound on the causal effect of academic achievement at age 16 on dropout by age 21. Second, any measurement error in our measure of short-term academic achievements will attenuate the indirect effects of teacher absence. Finally, teacher absence has larger effects on the academic achievements of low achievers, as shown by the quantile regressions, *and* the likelihood of dropout for low achievers is more influenced by their academic achievements than for high achievers (i.e. nonlinearity in both treatment-mediator association and mediator-outcome association) (Appendix Figure A8).

Because of these challenges, we can only provide a rough calculation of how much of the long-term effects of teacher absence on dropout that operates via shorter-term academic achievements.^7^ Using the product method to map the effects of teacher absence *on grades* in different parts of the grade distribution (same-sex twin fixed effects) to the effects *of grades* on dropout across the grade distribution (quantile regression estimates), our calculations suggest that the effect on academic achievements explains about one third (36%) of the effect of teacher absence on future school dropout (Appendix Figure A9).^8^

## Discussion

Teacher absence has been shown to harm student achievement (Miller *et al*. 2008; Clotfelter *et al*. 2009; Herrmann and Rockoff 2012). However, the literature is scarce, consisting of only a handful of US studies credibly identifying short-term effects in grades 4–8. This paper contributes to the literature by investigating whether socioeconomic backgrounds may compensate for reductions in instructional quality and whether teacher absence effects persist over time. We find robust evidence that teacher absence during lower secondary school (grades 8–10) in Norway impairs academic achievements in 10th grade. On average, an increase of about five percentage points in teacher absence – roughly equivalent to comparing a student at the 90th percentile of the teacher absence distribution with a student at the 10th percentile – reduces examination grades by 2.3% of a standard deviation. This effect size is on par with the previous US results in grades 4–8, which suggests that ten days of absence (roughly equal to a difference of 5.3 percentage points) reduce mathematics scores by between 1.2% and 3.2%, reading by 0.9%, and English by 0.6% (Miller *et al*. 2008; Clotfelter *et al*. 2009; Herrmann and Rockoff 2012). Given the similarity in teacher absence effects in the United States and Norway, where institutional contexts differ, we expect similar-sized teacher absence effects in other European countries.

We believe our study is the first to identify the long-term effects of teacher absence, finding that teachers’ absence in lower secondary school (ages 13–16) increases the likelihood of school dropout by age 21. Teacher absence depresses the lower achievers’ grades more than it does for the high achievers, suggesting stronger implications for their likelihood of later school dropout, since they are more likely to be at the margin for other reasons. In line with this, we find that the adverse effects of teacher absence on academic achievements in the lower end of the grade distribution translate into a sizeable effect on school dropout. An increase in teacher absence by five percentage points increases the likelihood of school dropout five years after completion of lower secondary school by 0.6 percentage points. This average effect size corresponds to a 2.1% increase in the dropout rate.

When teacher absence affects students’ educational outcomes, teacher absence is also likely to influence social inequality (Downey and Condron 2016). Teacher absence can disproportionally harm disadvantaged students in two major ways. First, *exposure* to teacher absence can be higher in schools that serve disadvantaged students. Second, the *effect* can be larger for disadvantaged students. On exposure, we find that teacher absence in Norway is only weakly related to the students’ socioeconomic and immigrant background. This equal exposure by socioeconomic background contrasts evidence from the United States (Clotfelter *et al*. 2009), reflecting a modest degree of school and neighborhood segregation in Norway (Tammaru *et al*. 2015; Hermansen *et al*. 2020).

Despite negligible differences in teacher absence exposure by parental SES, our evidence shows that teacher absence contributes to social inequality in long-term educational attainment. Disadvantaged children bear most of the burden of teacher absence; for example, an increase in teacher absence of 5 percentage points increases the likelihood of dropout for students of low-income parents by about one percentage point. In contrast, the dropout rate is unaffected by teacher absence for students with high-income parents. This heterogeneous effect of teacher absence translates into an impact on the socioeconomic dropout gap. In a hypothetical world without teacher absence, the gap between high-income and low-income students may be over 4% smaller (Appendix Table A9). This reduction is sizeable given that it is the effect of exposure over three school years only and that teacher absence is merely one of several contextual factors that may influence students’ dropout rates.

This paper adds to burgeoning literature demonstrating the importance of the school environment for social inequality, even in relatively egalitarian school systems. For example, recent studies have shown that average estimates conceal that school effects may be stronger for some groups, such as children at risk of poor achievement (Cheesman *et al*. 2022b; Cheesman *et al*. 2022a). Moreover, it has been shown that students in the same schools may face different environments due to the within-school clustering of friendship networks (Engzell and Raabe 2022; Chetty *et al*. 2022). This paper highlights that despite similar exposure to teacher absence, socioeconomic differences in the effects of teacher absence result in long-lasting social inequality in dropout rates. Thus, studying the heterogeneous impacts of contextual exposures is needed to understand schools’ role in shaping inequality.

Overall, the strong socioeconomic gradient in the effects of teacher absence could suggest that teacher absence induces a compensatory response from high-resource parents. That would be compatible with what is found in studies of compensatory advantage, such as birth weight effects (Torche and Echevarría 2011), sibship size effects (Tanskanen *et al*. 2016), and month of birth effects (Bernardi and Grätz 2015; Bernardi 2014). However, the socioeconomic gradients are compatible with other explanations too. To begin, if school and family inputs are substitutes, diminishing returns to total inputs imply that variation in teacher absence has less impact on students from high socioeconomic backgrounds compared to disadvantaged children. Further, the socioeconomic gradients in the effects of teacher absence can also be explained by disadvantaged students at risk of academic failure being more dependent on positive student-teacher relationships (Crosnoe *et al*. 2004) and being more sensitive to disruptions in the learning environment (Borman and Kimball 2005).

Despite several strengths, including being able to follow students into adulthood and discover differential effects by parental backgrounds, our study has limitations that must be considered. We use a school fixed effects model to account for factors influencing both student outcomes and teacher absence. We cannot prove beyond all doubt that other unobserved concurrent changes occurring within schools are not driving our results, but the robustness checks suggest that our identification strategy is sound. Furthermore, lacking individual student-teacher matches, we aggregate teacher absence at the school level. Although we argue that this aggregation leads to less precision and *not* the attenuation bias one might expect (see Online Appendix C), we cannot test this empirically. Moreover, our teacher absence measure is limited because it excludes short absence spells (<16 days), which means we can only study the effects of long-term teacher absence. Additionally, if shorter-term absence spells correlate with longer-term spells, this correlation may lead to an upward bias in our estimates (see Online Appendix D).

In conclusion, we have shown that teacher sickness absence has long-term effects on school dropout for students from lower socioeconomic backgrounds, while having nearly no long-term effect for students from upper socioeconomic backgrounds. This finding demonstrates that reductions in instructional quality increase social inequality in long-term educational outcomes. Further research should unravel the mechanisms driving these heterogeneous effects, to increase our understanding of processes that lead to social inequality, and inform policy that can compensate for disadvantages caused by teacher absence and other reductions in instructional quality.

## Research ethics statement

The data collected in this article is in accordance with legislation and approved by the Norwegian Data Protection Authority. The research is conducted in line with ethical guidelines set by in The National Committee for Research Ethics in the Social Sciences and the Humanities (NESH) in Norway. The Personal Data Act in Norway makes exceptions from informed consent with regard to the use of existing register data because it is not feasible to obtain consent from all persons whom the registers cover.

## Supplementary Material

Supplemental Material

## Footnotes

1 Teacher absence rates in developing countries are considerably higher, with for example rates in the range of 20–44% begin reported in Kenya and India (Clotfelter et al. 2009).

2 A randomized experiments in India demonstrated that reducing teacher absence in India 21 percentages points improved children’s test scores by 0.17 standard deviation (Duflo et al. 2012).

3 Miller et al. (2008) also identify the local average treatment effect of (unplanned) teacher absence using the number of frigid days and number of days with a snow-pack as instrumental variables. The IV estimate is in the same direction, but 15 times larger than the OLS estimate.

4 There is some evidence, however, suggesting a positive interaction effect of family and school resources. Kim (2001) finds that low-educated parents cut back on their childcare time use in response to an increase in per pupil school expenditure. Highly educated mothers, however, keep their childcare time fixed. When low-educated parents respond, teacher absence may be more harmful for children with highly educated parents, consistent with evidence of more adverse effects of parental divorce for these children (Bernardi and Radl 2014).

5 An ongoing discussion on which quantile regression approach should be used to estimate QTEs is currently taking place (Wenz 2019; Killewald and Bearak 2014; Rios-Avila and Maroto 2020; Borgen et al. 2023). In our case, the coefficients are largely indistinguishable whether we use the RQR model or the generalized quantile regression (GQR) model of Powell (2020), both of which are developed to identify QTEs. Moreover, the overall pattern is similar when using the unconditional quantile regression model (Firpo et al. 2009), although the coefficients differ somewhat from the RQR and GQR models (Appendix Figure A6).

6 The heterogeneous effects across the outcome distribution is even more pronounced when looking at grade point average from upper secondary education, which includes all teacher-assessed grades and examination grades (Appendix Figure A5).

7 We cannot use twin fixed effects to examine teacher absence effects because there is almost no variation in teacher absence within pairs of twins. Thus, controlling for academic achievements in a regression of dropout on teacher absence (i.e., the difference method to mediation) will introduce a collider bias (Elwert and Winship 2014; VanderWeele 2015).

8 If we ignore non-linearity and simple calculate the product of the teacher absence effect on examination grades (Table 2) and the examination grades effect on dropout in a same-sex twin fixed effects model (Table A8), we find that academic achievements mediate about 30% of the association between teacher absence and school dropout (−0.4612∗−0.0725=0.0334). Note that mediation methods will tend to exaggerate the achievement channel when the dropout equation’s exam coefficient is upward biased. Suppose we use the exam coefficient from the school fixed effects model in Table A8 instead of the twin fixed effects model. In that case, 64% is mediated by achievement. Likewise, controlling for examination grades in a regression of dropout on teacher absence (i.e., the difference method) suggests that 58% of the variation is mediated by achievement, and the teacher absence effect is no longer significant at conventional levels (not shown).
