# Supplementary material for: Socioeconomic differences in the long-term effects of teacher absence on student outcomes
Source: Eur Soc. 2023 May 20;26(3):639–67. doi: 10.1080/14616696.2023.2212744 (PMC11262431; doi:10.1080/14616696.2023.2212744)
Supplement: Supplemental Material [file REUS_A_2212744_SM7266.pdf]

Supplementary online appendix

Socioeconomic differences in the long-term effects of teacher absence on student outcomes

Nicolai Topstad Borgen[1,2,3,4], Simen Markussen[5], and Oddbjørn Raaum[5]

[1] Department of Special Needs Education, University of Oslo, Oslo, Norway
[2] Center for Research on Equality in Education, University of Oslo, Oslo, Norway
[3] Department of Sociology and Human Geography, University of Oslo, Oslo, Norway
[4] Centre for the Study of Professions, Oslo Metropolitan University, Oslo, Norway
[5] Ragnar Frisch Centre for Economic Research, Oslo, Norway

Content of the Supplementary Online Appendix:

Appendix A: Supplementary tables and figures
Appendix B: Robustness checks
Appendix C: School Aggregation
Appendix D: Bias by short-term teacher absence spells
Appendix E: Replication files

Online Appendix A: Supplementary tables and figures

Table A1: Teacher absence by year

	Mean	SD	5th	10th	25th	50th	75th	90th	95th
2002	0.043	0.020	0.015	0.020	0.029	0.041	0.054	0.068	0.075
2003	0.044	0.021	0.016	0.019	0.030	0.041	0.055	0.071	0.079
2004	0.050	0.030	0.017	0.024	0.034	0.045	0.062	0.079	0.091
2005	0.052	0.031	0.018	0.023	0.033	0.048	0.063	0.080	0.094
2006	0.054	0.034	0.017	0.023	0.035	0.047	0.065	0.088	0.105
2007	0.050	0.024	0.017	0.023	0.034	0.047	0.063	0.080	0.093
2008	0.053	0.025	0.018	0.024	0.037	0.048	0.067	0.083	0.099
2009	0.053	0.025	0.019	0.025	0.036	0.048	0.066	0.088	0.102
2010	0.052	0.025	0.020	0.025	0.034	0.047	0.063	0.087	0.103
2011	0.049	0.024	0.018	0.024	0.033	0.045	0.059	0.080	0.097
2012	0.045	0.021	0.018	0.023	0.030	0.042	0.054	0.072	0.086
2013	0.043	0.019	0.018	0.022	0.029	0.040	0.052	0.065	0.077
2014	0.040	0.017	0.016	0.021	0.027	0.039	0.049	0.061	0.067
2015	0.039	0.016	0.014	0.020	0.028	0.038	0.048	0.059	0.065
Total	0.048	0.025	0.017	0.022	0.032	0.044	0.059	0.075	0.091

Table A2: Correlation between teacher absence rates in grades 8, 9, and 10.

	8th grade	9th grade	10th grade
8th grade	1		
9th grade	0.221***	1	
10th grade	0.162***	0.236***	1

* $p < 0.05$, ** $p < 0.01$, *** $p < 0.001$

Table A3: Effects of teacher absence on grade point average from lower secondary school.

	(1)<br>Grade point average
Teacher absence	-0.2459**
	(0.0950)
Control	Yes
Teacher	Yes
Observations	540780

Note: Standard errors clustered at schools in parentheses. All models with school fixed effects, individual controls, and teacher controls.
* $p < 0.05$, ** $p < 0.01$, *** $p < 0.001$

Table A4: Effects of teacher absence on examination grades using RQR

	(1) 10th percentile	(2) 25th percentile	(3) 50th percentile	(4) 75th percentile	(5) 90th percentile
Teacher absence	-0.8697***	-0.5933**	-0.4037*	-0.4170**	-0.3425*
	(0.2218)	(0.1851)	(0.1959)	(0.1500)	(0.1491)
Individual controls	Yes	Yes	Yes	Yes	Yes
Teacher controls	Yes	Yes	Yes	Yes	Yes
Difference in coefficients					
vs. 10th		-0.2765*	-0.4661**	-0.4527*	-0.5272*
		(0.1281)	(0.1711)	(0.2033)	(0.2230)
vs. 25th	0.2765*		-0.1896	-0.1763	-0.2508
	(0.1281)		(0.1069)	(0.1488)	(0.1766)
vs. 50th	0.4661**	0.1896		0.0133	-0.0612
	(0.1711)	(0.1069)		(0.1054)	(0.1515)
vs. 75th	0.4527*	0.1763	-0.0133		-0.0745
	(0.2033)	(0.1488)	(0.1054)		(0.1215)
vs. 90th	0.5272*	0.2508	0.0612	0.0745	
	(0.2230)	(0.1766)	(0.1515)	(0.1215)	
Observations	534078	534078	534078	534078	534078

Note: Standard errors clustered at schools in parentheses. All models with school fixed effects, individual controls, and teacher controls. The difference in coefficients shows difference between coefficients at different quantiles, along with the standard error of the difference.
* $p < 0.05$, ** $p < 0.01$, *** $p < 0.001$

Table A5: Including teacher absence measured during grades 8, 9, and 10.

	Examination grades				Dropout			
	(1)	(2)	(3)	(4)	(5)	(6)	(7)	(8)
Grade 8	-0.1515*	-0.1559*			0.0309	0.0269		
	(0.0763)	(0.0775)			(0.0221)	(0.0224)		
Grade 9	-0.2042***		-0.2246***		0.0281		0.0250	
	(0.0610)		(0.0630)		(0.0227)		(0.0226)	
Grade 10	-0.0997			-0.1140	0.0531*			0.0520*
	(0.0729)			(0.0763)	(0.0222)			(0.0222)
Observations	534078	534707	538727	541491	346551	347198	350457	352579

Note: Standard errors clustered at schools in parentheses. All models with school fixed effects, individual controls, and teacher controls.
* $p < 0.05$, ** $p < 0.01$, *** $p < 0.001$

Table A6: Mean earnings rank at age 32-36 by educational attainment.

	(1)<br>Earnings rank
Lower secondary education or lower	37.3977***
	(0.0684)
Upper secondary education	50.6056***
	(0.0619)
Higher education	60.3919***
	(0.0646)
Observations	529700

Note: Sample of parents born between 1965 and 1975. Education at age 30.
Standard errors in parentheses
* $p < 0.05$, ** $p < 0.01$, *** $p < 0.001$

Table A7: Teacher absence exposure by parental background of the student.

	(1)<br>Teacher absence
Panel A: Parental earnings rank	
1-24th Percentile	Ref.
25-49th Percentile	-0.0002*
	(0.0001)
50-74th Percentile	-0.0002
	(0.0001)
75-99th Percentile	-0.0008***
	(0.0001)
Constant	0.0478***
	(0.0001)
Observations	555280
Panel B: Parental education	
Lower sec. educ.	Ref.
Upper sec. educ.	-0.0003**
	(0.0001)
Higher educ.	-0.0006***
	(0.0001)
Constant	0.0480***
	(0.0001)
Observations	554616

Note: Standard errors in parentheses. See Table 3 for description of parental earnings and parental education.
* $p < 0.05$, ** $p < 0.01$, *** $p < 0.001$

Table A8: Effects of examination grades on school dropout.

	(1) Dropout	(2) Dropout	(3) Dropout	(4) Dropout	(5) Dropout
Examination grades	-0.1544***	-0.1545***	-0.1122***	-0.0838***	-.0725***
	(0.0007)	(0.0012)	(0.0028)	(0.0121)	(0.0144)
Individual controls	Yes	Yes	Yes		
School fixed effects		Yes			
Siblings fixed effects			Yes		
Twin pair fixed effects				Yes	
Same-sex twin pair fixed effects					Yes
Observations	352636	351813	352636	8080	5593

Note: Siblings are defined as individuals with the same biological mother. Standard errors in parentheses, with conventional standard errors in (1), standard errors clustered at schools in (2), and standard errors clustered by mother in (3)-(5).

* $p < 0.05$, ** $p < 0.01$, *** $p < 0.001$

Table A9: Percent reduction in the socioeconomic dropout gap in a hypothetical setting without teacher absence.

	Observed dropout rate ($\bar{Y}$)	Teacher absence effect (from Table 2) (δ)	Average teacher absence (TS)	Hypothetical dropout without absence
Low-income children	.4480	.1942	.0502	.4383
High-income children	.1568	-.0521	.0504	.1594

Note: Hypothetical dropout rate without absence is calculated as: $\bar{Y} - \delta * TS$. The observed gap is .291, while the hypothetical dropout gap without absence is .2789. The percent reduction is calculated as $\left(1 - {.2789}/{.291}\right) * 100 = 4.2$.

Table A10: Variation in teacher absence and outcomes within and between schools.

	Mean	Standard deviations		
		Total	Between schools	Within schools
Teacher absence	.0476	.0247	.0241	.0194
Examination grades	.0297	.995	.3799	.9741
School dropout	.2859	.4519	.2388	.4453

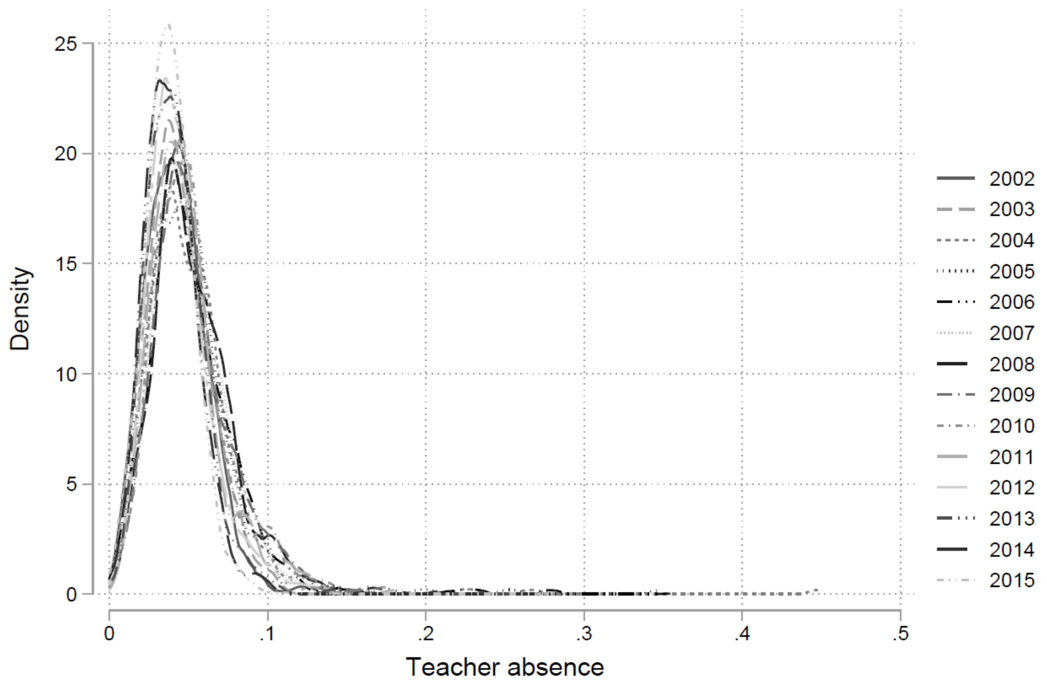

Figure A1: Density of teacher absence by year of graduation.

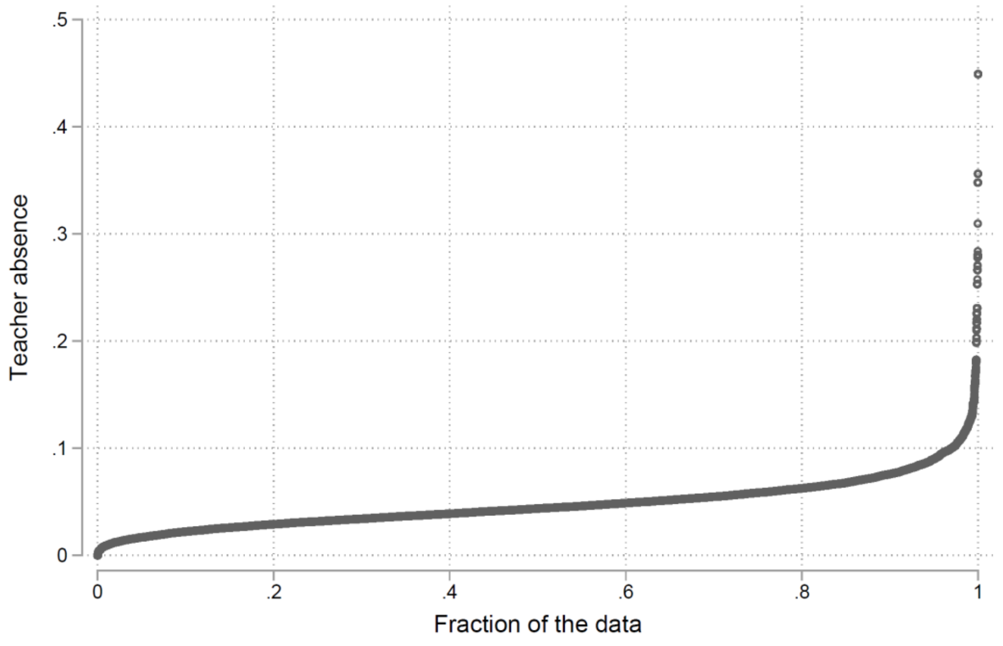

Figure A2: Quantile-plot showing the fraction of data with teacher absence at a given level.

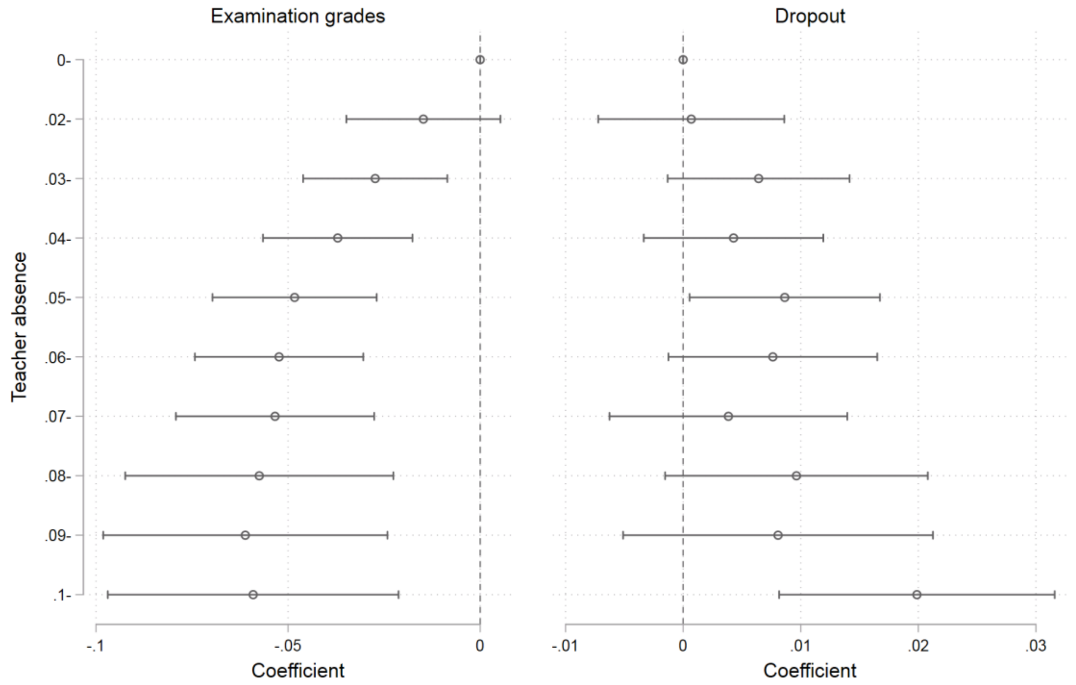

Figure A3: Non-linear effects of teacher absence on dropout and examination grades with 95% CI.

Note: The teacher absence variable is grouped in 10 groups and included as dummies in a school fixed effects model.

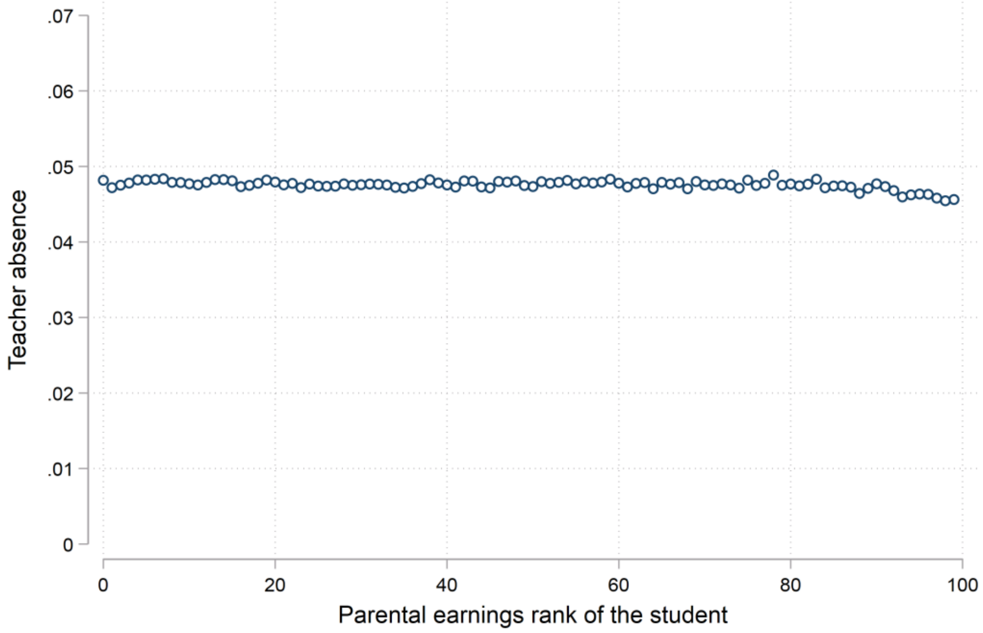

Figure A4: Bivariate association between parental earnings rank of the student and teacher absence.

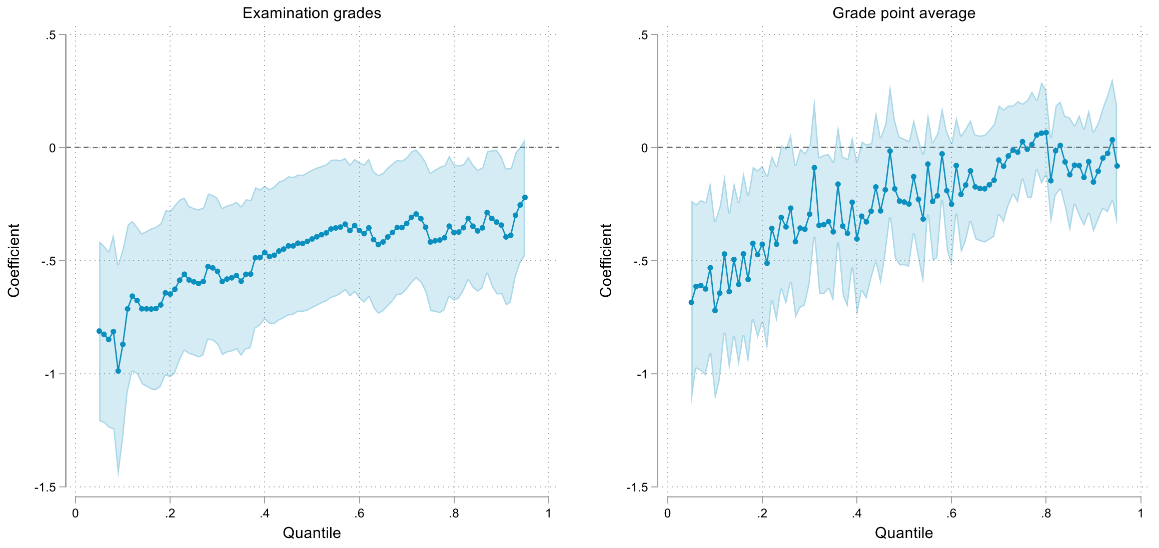

Figure A5: Effects of teacher absence on examination grades and grade point average across the outcome distributions.

Note: Coefficients are estimated using RQR. Examination grades are artificially smoothed but not grade point average.

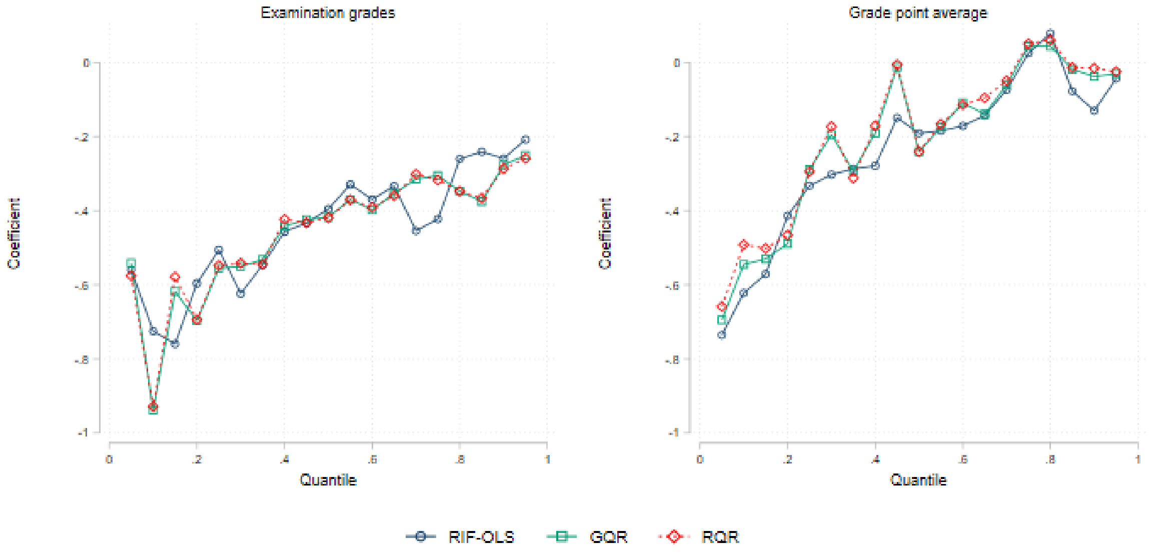

Figure A6: Effects of teacher absence on examination grades and grade point average using RQR, generalized quantile regression (GQR), and unconditional quantile regression (RIF-OLS).

Note: In RIF-OLS, the kernel density estimate is oversmoothed for examination grade because of heaping, but not for grade point average. To smooth the examination grades variable in GQR and RQR, we add uniform noise to jitter the data (Machado and Silva 2005) using a uniform distribution over the interval [-0.5, 0.5]. The GQR model is estimated using the user-written Stata command genqreg with dummies for school ID.

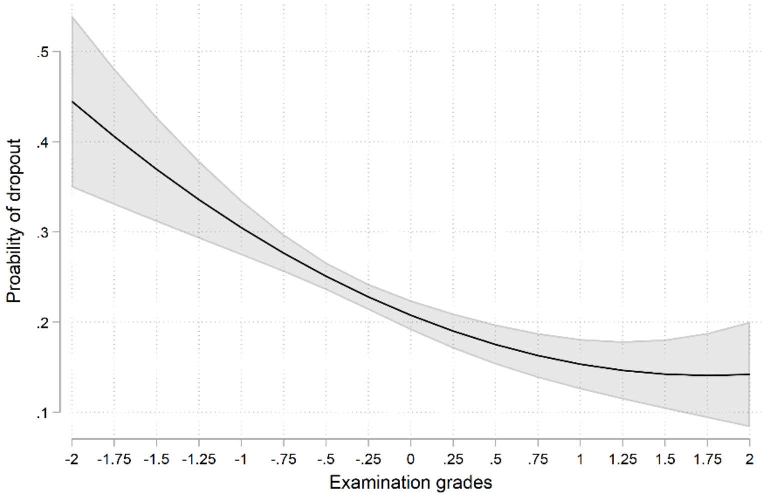

Figure A8: Association between examination grades and dropout in a same-sex twin fixed effects model.

Note: The predicted probability of dropout is based on a linear probability model that includes examination grades, examination grades squared, same-sex twin pair fixed effects, and cohort fixed effects. Twins are defined as individuals born in the same calendar month by the same biological mother. Standard errors clustered at mothers. See Appendix Table A8 for estimated association between examination grades and dropout without twin pair fixed effects.

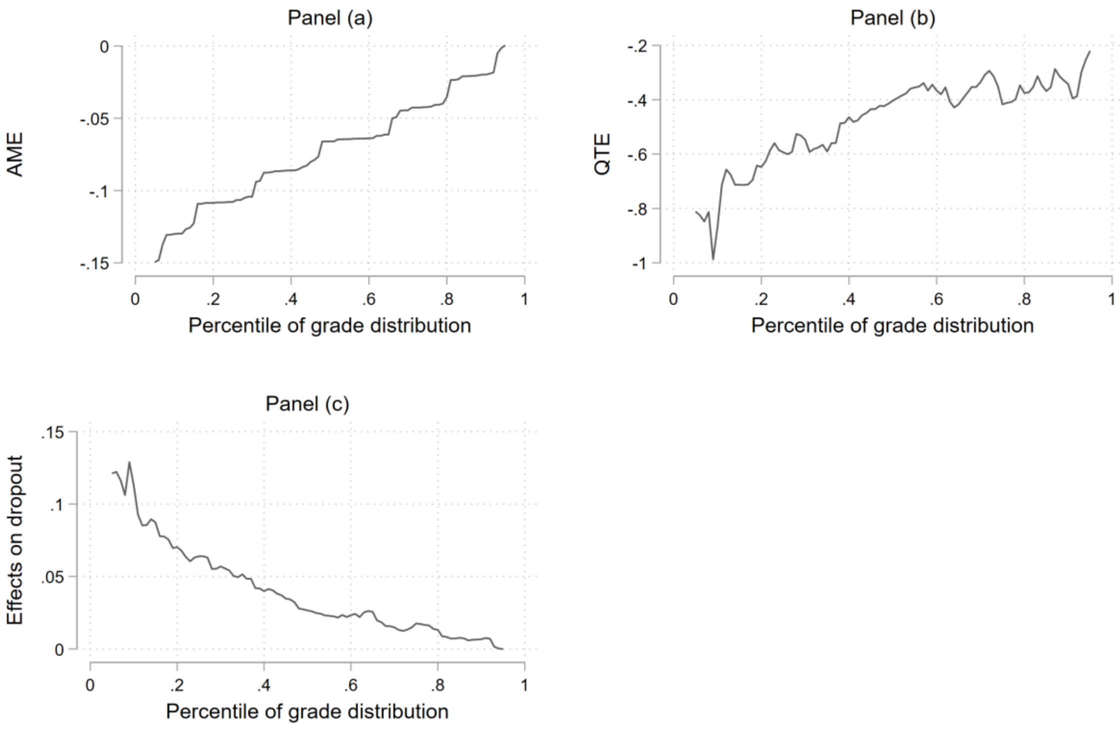

Figure A9: Product method to suggest how much of the teacher absence effects on dropout is explained by examination grades.

Note: Panel (a) display the average marginal effects (AME) calculated based on a linear probability model of the effects of examination grades and its squared term on school dropout, using a sample of same-sex twins with a twin pair fixed effect and cohort fixed effects. Subsequently, AME is calculated at various percentiles of the grade distribution. Panel (b) includes the quantile treatment effects, and is identical to the estimates shown in Figure 2. Panel (c) display the product of the coefficients in Panel (a) and Panel (b), and shows the calculated effects of teacher absence on dropout – mediated through examination grades – for students in different part of the grade distribution. The average of the calculated effects of teacher absence on dropout in panel (c) is 0.0399.

Online Appendix B: Robustness checks

When concluding on cause-and-effect relationships, it is crucial to account for factors that may confound the estimates. Based on the school fixed effects model, we find that teacher absence impairs students' academic achievements and subsequently increases school dropout risk. Taking account of time-invariant school characteristics (i.e., school fixed effects) is seemingly important as the estimated effects of teacher absence without these fixed effects are considerably larger (Table 2). Still, a concern is that the within-school variation in teacher absence could be (partly) caused by variation in the share of students with behavioral and academic problems, even if we condition on time-variant student family background characteristics. This online appendix investigates this concern empirically by using a subset of the overall cohorts, for whom we have access to richer data. The smaller sample size in most of these sensitivity analyses reduces the precision of the estimated effects. For that reason, we choose to focus on the average impact of teacher absence.

We explore the robustness of the results from three complementary angles: by studying whether potential confounders influence teacher absence (i.e., with teacher absence as an outcome, Table B1), whether teacher absence influences potential confounders (Table B2), and by comparing the estimated effects of teacher absence with and without the potential confounder (Tables B3-B4). The benefit of the first two approaches is that they examine the selection mechanisms we are interested in, and we need to be less concerned about whether the confounders are adequately measured. The latter approach's advantage is that it directly suggests the size of bias in the teacher absence coefficient, but measurement error or incompleteness in the observed proxy matters. As an illustration, child delinquents' classroom behavior may influence teachers' absence, allowing us to test the concern of reverse causality. Still, as child delinquency is rare, it is a poor proxy for general classroom behavior, and controlling for child delinquency is unlikely to influence estimates notably.

Table B1: Variation in teacher absence

	(1)<br>Teacher absence
Panel A:	
Pre-treatment test score	0.000053
(Mean=0.017, SD=0.883)	(0.000058)
Observations	238133
Panel B:	
Charged by age of 12	0.000176
(Mean=0.010, SD=0.098)	(0.000300)
Observations	549916
Panel C:	
Poor school behavior by end of 10th grade	0.000136
(Mean=0.023, SD=0.149)	(0.000357)
Observations	235430
Panel D:	
Students' GP visits grade 8-10	0.000014**
(Mean=6.672, SD=6.620)	(0.000005)
Observations	213387

Note: Standard errors clustered at school level in parentheses. All models with school fixed effects, individual controls, and teacher controls. Estimates in panels are from different models.
* $p < 0.05$, ** $p < 0.01$, *** $p < 0.001$

Table B2: Left-hand side balancing test.

	(1)<br>Pre-treatment test scores	(2)<br>Charged by age of 12	(3)<br>Poor school behavior by end of 10th grade	(4)<br>Students' GP visits grade 8-10
Teacher absence	0.1898	0.0048	0.0141	3.1082**
	(0.2033)	(0.0080)	(0.0368)	(1.1255)
Observations	238133	549916	235430	213387

Note: Standard errors clustered at school level in parentheses. All models with school fixed effects, individual controls, and teacher controls.
* $p < 0.05$, ** $p < 0.01$, *** $p < 0.001$

Table B3: Robustness checks

	Value-added model		Common health shock controls		Behavioral problems controls							
	Examination grades		Examination grades		Examination grades				School dropout			
	(1)	(2)	(3)	(4)	(5)	(6)	(7)	(8)	(9)	(10)	(11)	(12)
Teacher absence	-0.6881*	-0.8454***	-0.8922***	-0.8723***	-0.4625**	-0.4620**	-0.7317**	-0.7212**	0.1109**	0.1105**	0.2619*	0.2597*
	(0.2835)	(0.2474)	(0.2521)	(0.2533)	(0.1431)	(0.1434)	(0.2384)	(0.2283)	(0.0361)	(0.0363)	(0.1266)	(0.1275)
Pre-academic achievement		0.6795***										
		(0.0024)										
Students GP visits grade 8-10				-0.0082***								
				(0.0004)								
Criminal charges by age 12						-0.3604***				0.1650***		
						(0.0127)				(0.0078)		
Poor school behavior grade 10								-0.8300***				0.3901***
								(0.0137)				(0.0081)
Observations	232193	232193	205267	205267	533986	533986	228693	228693	346551	346551	117148	117148

Note: Standard errors clustered at school level in parentheses. All models with school fixed effects, individual controls, and teacher controls. Sample size varies because of data availability.
* $p < 0.05$, ** $p < 0.01$, *** $p < 0.001$

Table B4: Robustness checks

	Examination grades					School dropout			
	(1)	(2)	(3)	(4)	(5)	(6)	(7)	(8)	(9)
Teacher absence	-0.5955**	-0.5814**	-0.5873**	-0.5922**	-0.5773**	0.2405*	0.2531*	0.2406*	0.2524*
	(0.1910)	(0.1893)	(0.1896)	(0.1906)	(0.1885)	(0.1006)	(0.0994)	(0.0989)	(0.0992)
Classroom noise grade 10		-0.1124***			-0.0886***				
		(0.0155)			(0.0157)				
School wellbeing grade 10			0.1280***		0.1066***		-0.0411***		-0.0388***
			(0.0146)		(0.0166)		(0.0078)		(0.0084)
School bullying grade 10				-0.3048***	-0.0216			0.0913**	0.0203
				(0.0680)	(0.0754)			(0.0297)	(0.0323)
Observations	305512	305512	305512	305512	305512	154972	154972	154972	154972

Note: Standard errors clustered at school level in parentheses. All models include school fixed effects, individual controls, and teacher controls. Measures of school environment are obtained from an annual nation-wide survey among all 10th graders in Norway (>90% response rate), which we can match to our register data on the school-cohort level for cohorts 2007-2012 and cohorts 2014-2015. Student-reported frequency of classroom noise is measured on a five-point scale (1=fully agree with classroom order, 5=fully disagree with classroom order), and is standardized to have a pupil-weighted mean of zero and standard deviation of 1. Student-reported school wellbeing is measured on a five-point scale (1=does not enjoy school very much, 5=enjoy school very much), and is standardized to have a pupil-weighted mean of zero and standard deviation of 1. School bullying measures the share of students that report being bullied at least 2-3 times a month, which in our sample is 7.9 percent of the students. For the cohorts 2007-2009, we do not observe the indicator classroom noise, and replace missing values with zero for these cohorts and include a dummy for missing values.
* $p < 0.05$, ** $p < 0.01$, *** $p < 0.001$

From 2007 and onwards, every student in Norway must undergo nationwide standardized testing in reading, math, and English. The 8th-grade test is basically an entry test since it occurs during the fall semester, shortly after the students start. Using the average of these national tests across subjects as a predictor, we do not find any indications of higher teacher absence in cohorts (within schools) with lower entry-test scores (panel A in Table B1, column 1 in Table B2). This suggests that reverse causality or simultaneity bias is not a major concern in our school fixed effects model, which aligns with previous literature using teacher fixed effects (Herrmann and Rockoff 2012).

Further, we test the concern of reverse causality and simultaneity bias more explicitly in Table B3, where we re-estimate our main results for a subset of the data (for which 8th-grade test scores are available) using a value-added school fixed effects model. Relative to the coefficients' precision, the point estimates are similar whether entry tests are controlled for or not; the coefficient difference between columns (1) and (2) is about 60% of the standard error. Finally, our identifying assumption is supported by 8th-grade test scores as a placebo outcome since there is no significant correlation between these scores and subsequent teacher absence for the same cohort (Appendix Table B5).

Table B5: Placebo outcome.

	Pre-treatment test score (1)
Teacher absence	0.1898
	(0.2033)
Observations	238133

Note: Standard errors clustered at school level in parentheses. All models with school fixed effects, individual controls, and teacher controls.
* $p < 0.05$, ** $p < 0.01$, *** $p < 0.001$

There is a potential concern that student behavior or an unhealthy school environment causes teacher absence and poor student performance. About 1 percent of the students have

received a criminal charge before entering lower secondary school (by the age of 12), which can be taken as an indicator of child behavioral problems.[1] We find that the rate of teacher absence is similar for students with criminal charges by the age of 12 as for other students (Panel B in Table B1, column 2 in Table B2). It is also reassuring that we find no association between our teacher absence measure and poor marks in order and conduct in grade 10 (Panel C in Table B1, column 3 in Table B2). In our sample, 2.3% of the students obtain poor marks in order and conduct, which measures behavior such as being late to class, not doing the homework, being violent, and cheating on tests. As this is graded in the last year of lower secondary schools, any positive association could be interpreted as a combination of a causal effect of teacher absence on behavioral problems *and* a causal effect of behavioral problems on teacher absence. Finding no association suggests that confounding because of time-variant (unobserved) student behavioral problems is not a major issue in the school fixed effects models. Again, we test this more explicitly in Table B3, and the inclusion of behavioral problem indicators does not change the estimated effects of teacher absence (columns 5-12).

Finally, we explore the importance of an unhealthy school environment using student-reported school environment measures, available from an anonymous annual nationwide survey among all 10th graders in Norway for cohorts after 2007. We find that more classroom noise, less school well-being, and more school bullying are associated with poorer examination grades and more dropout (Table B4). However, controlling for these variables does not change the estimated effects of teacher absence, leaving us less concerned about confounding school factors.

Besides reverse causality, another concern in the school fixed effects model is a common shock that increases teacher absence and directly affects students' learning. For some student cohorts, we can observe their visits to their general practitioner (GP) from the register of Control

[1] These criminal charges do not result in stays in a juvenile detention center, but the children are likely to be transferred to the child welfare services.

and Payment of Health Reimbursement (Torvik et al. 2018). Under the assumption that the variation in student GP visits within schools is a proxy variable for common health shocks, these GP visits could indicate to what extent such shocks bias our main results. Looking at the results in panel D of Table B1 and column 4 in Table B2, we see that the number of GP visits is weak but positively associated with teacher absence. However, the effect is trivial and could reflect a causal effect of teacher absence on student health: a 1SD increase in the number of GP visits increases the teacher absence rate by 0.00009 (Table B1), and a five-percentage point increase in teacher absence raises GP visits by 0.05 per year (Table B2).[2] Moreover, the estimated effects of teacher absence on standardized examination grades are nearly identical with and without student GP visits as a control (columns 3-4 in Table B3).

Since dropout rates differ by region in Norway, we are concerned about correlated trends in the outcome variables and the treatment variable that are regionally specific. Thus, we have checked whether the results are robust to including regional-specific cohort fixed effects (19 counties). Concerning short-term academic achievements, the results are largely unaffected by the inclusion of school county-specific cohort fixed effects. The effects on dropout are more affected by county-specific cohort fixed effects, with point estimates reduced by roughly 20%.[3] However, the precision is also reduced when including regional-specific cohort fixed effects, and the confidence intervals with the different specifications overlap (Appendix Figure B1).

---

[2] Calculated as the effects of teacher absence multiplied by 5 (i.e., five percentage point increase) divided by 3 to get the annual effect: $3.1082 * .05/_3$.

[3] As shown in Online Appendix Figure B1, including school municipality specific cohort fixed effects for the 286 school municipalities reduces the estimated effects of teacher absence on dropout by roughly 35% and renders the coefficient significant at the 9.3% level only.

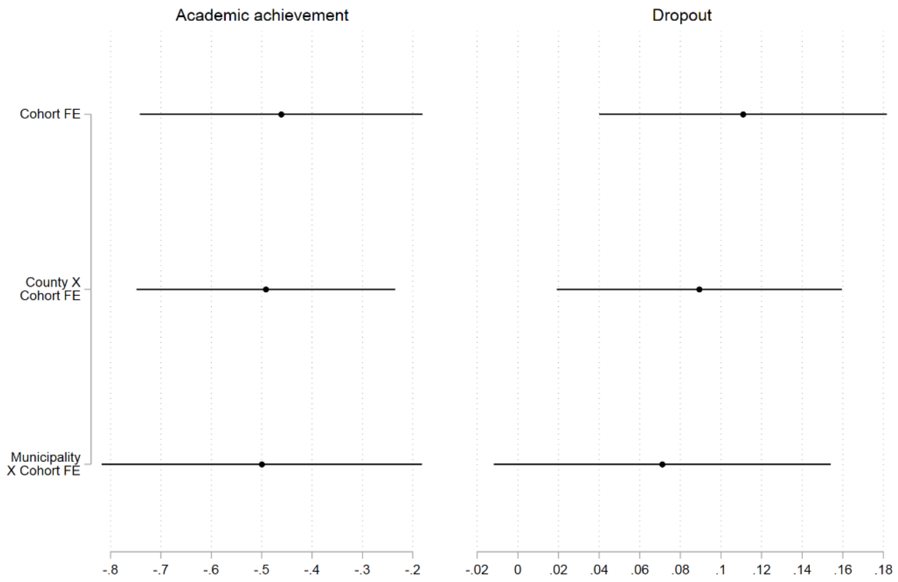

Figure B1: Effects of teacher absence controlling for cohort fixed effects, school county specific cohort fixed effects, and school municipality specific cohort fixed effects.

Note: The coefficient in the cohort fixed effects and school county specific cohort fixed effects are also shown in Table B6.

Table B6: Robustness checks including regional time trends for the 19 school counties.

	Examination grades		School dropout	
	(6)	(7)	(8)	(9)
Teacher absence	-0.4612**	-0.4917**	0.1109**	0.0893*
	(0.1430)	(0.1308)	(0.0361)	(0.0357)
County cohort FE		Yes		Yes
Observations	534078	532167	346551	344582

Note: Standard errors clustered at school level in parentheses. All models with school fixed effects, individual controls, and teacher controls.
* $p < 0.05$, ** $p < 0.01$, *** $p < 0.001$

Finally, the school fixed effects only account for *stable* between-school differences in teacher characteristics, student characteristics, and school resources. With a long observation window, the fixed effects estimator may produce biased estimates if schools systematically change over time in a way correlated with teacher absence. To meet this concern, we re-estimate the main results with a six-year rolling average, which for the latest cohorts coincides with the period we have information on entry tests (see above). The average teacher absence effect

fluctuates somewhat across the cohorts, but there is no trend (Panel A and B in Online Appendix Figure B2). In contrast, there seems to be a decline in the socioeconomic gradients in the teacher absence effects on examination grades towards the end of our observation period. Unlike for the first nine cohorts, the parental earnings interaction coefficient is close to zero in the last six cohorts, which holds whether entry test scores are conditioned on or not (Panel C). However, the confidence intervals overlap for all cohorts, and no strong conclusion concerning time trends can be drawn from the data.

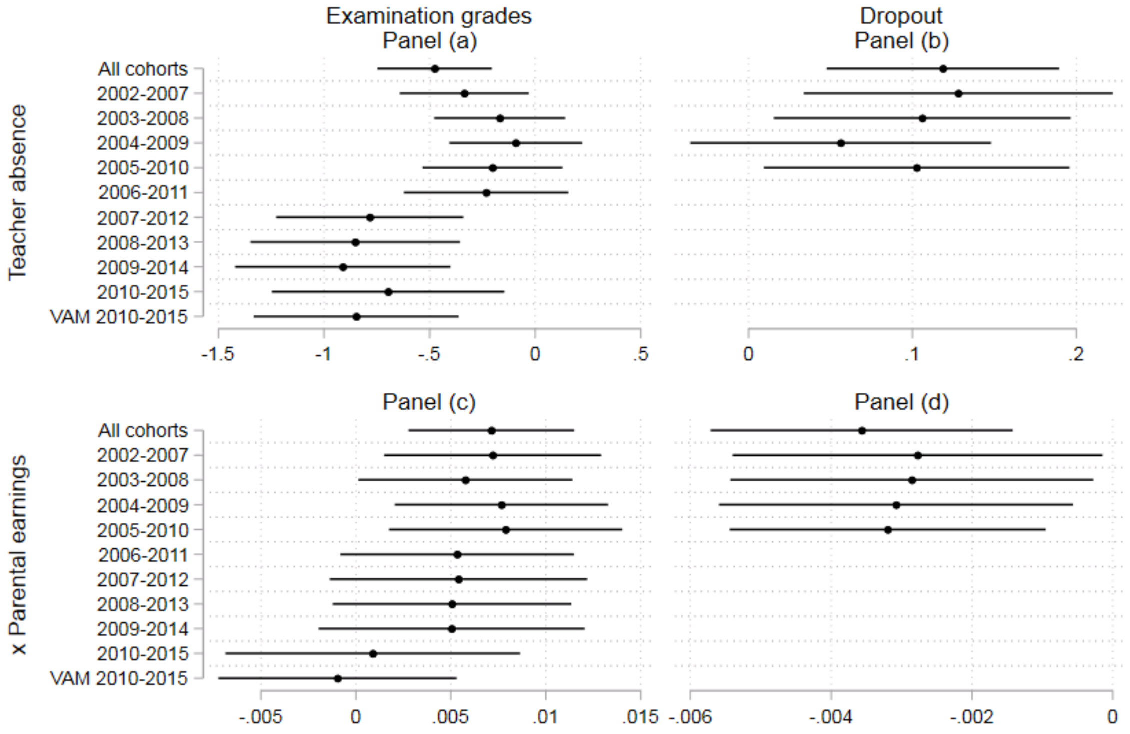

Figure B2: Socioeconomic gradients in teacher absence effects in six-year rolling averages compared to all available cohorts.

Note: The first row in each panel includes all available cohorts for the outcomes. The six-year rolling average estimates are estimated in separate regression analyses. The VAM model controls for the entry test score. The upper panels (a) and (b) show the main effect evaluated at the median of parental earnings, while the lower panels (c) and (d) show teacher absence interacted with ranked parental earnings. The parental earnings variable ranges from 0 (low parental earnings) to 99 (high parental earnings) and is centered at the median.

Online Appendix C: School aggregation

This appendix simulates data to show that replacing the true teacher absence rate (T_{sc}) with the error-ridden observed school-level teacher absence rate (TS_{sc}) produce consistent estimates of the true effect, only less precisely estimated. We refer to these two absence rate variables as true and observed teacher absence in this appendix. Stata code to replicate the simulation results is included at the end of this appendix.

We construct 1000 lower secondary schools s, each with eight school cohorts c of 20 individuals i observed from grades g 8 through 10. Then, we define teacher absence at the school-by-grade level as $T_{scg} = .05 + u_s * -0.01 + e_{scg}$, where $e_{scg} \sim Beta(0.90,20)$ and $u_s \sim N(0,1)$ is an unobserved time-invariant school quality variable that reduces teacher absence. The true treatment variable is defined as $T_{sc} = {T_{s8} + T_{s9} + T_{s10}}/_{3}$ and measure individuals' average exposure to teacher absence. The observed treatment variable is based on the school's average teacher absence across grades 8-10 $TS_{sc} = {TS_{sc8} + TS_{sc9} + TS_{sc10}}/_{3}$, where TS_{sc8} is the school average across grades 8-10 the year the cohort attended grade 8, TS_{sc9} is the school average the year the cohort attended grade 9, and TS_{sc10} is the school average the year the cohort attended grade 10. Finally, the outcome in grade 10 is defined as $y_{isc} = T_{sc} * -.10 + u_s + \varepsilon_{isc}$, where $\varepsilon_{isc} \sim N(0,1)$. The analysis data consists of one observation for each individual (in grade 10) (N=160,000), clustered in 1,000 schools and eight school cohorts.

Using a school fixed effects model, we compare the estimate of β_1 with that of δ_1 from the following two models across 10,000 independent draws:

$$y_{isc} = \beta_0 + \beta_1 T_{sc} + \alpha_s + \epsilon_{isc}$$

$$y_{isc} = \delta_0 + \delta_1 TS_{sc} + \alpha_s + \epsilon_{isc}$$

The results displayed in Table C1 and Figure C1 below demonstrate that replacing the true teacher absence rate with the average across school years produces the same coefficients. The coefficients of the true teacher absence and the error-ridden observed teacher absence are both close to -0.10, which is the true effect (Appendix Table C1). However, when using the observed teacher absence variable, the standard deviation across the random draws is twice as high as when using the true teacher absence variable, reflecting the fact that the observed teacher absence variable produces less precise estimates. The precision penalty is also shown graphically in Appendix Figure C1; the variation in the coefficients across the random draws is much higher with the observed teacher absence variable than the true teacher absence variable.

Appendix Table C1: Average coefficient and standard deviation across 10,000 draws

	**Coefficient**	**Standard deviation**
Observed teacher absence	-0.0995	0.2104
True teacher absence	-0.1016	0.1076

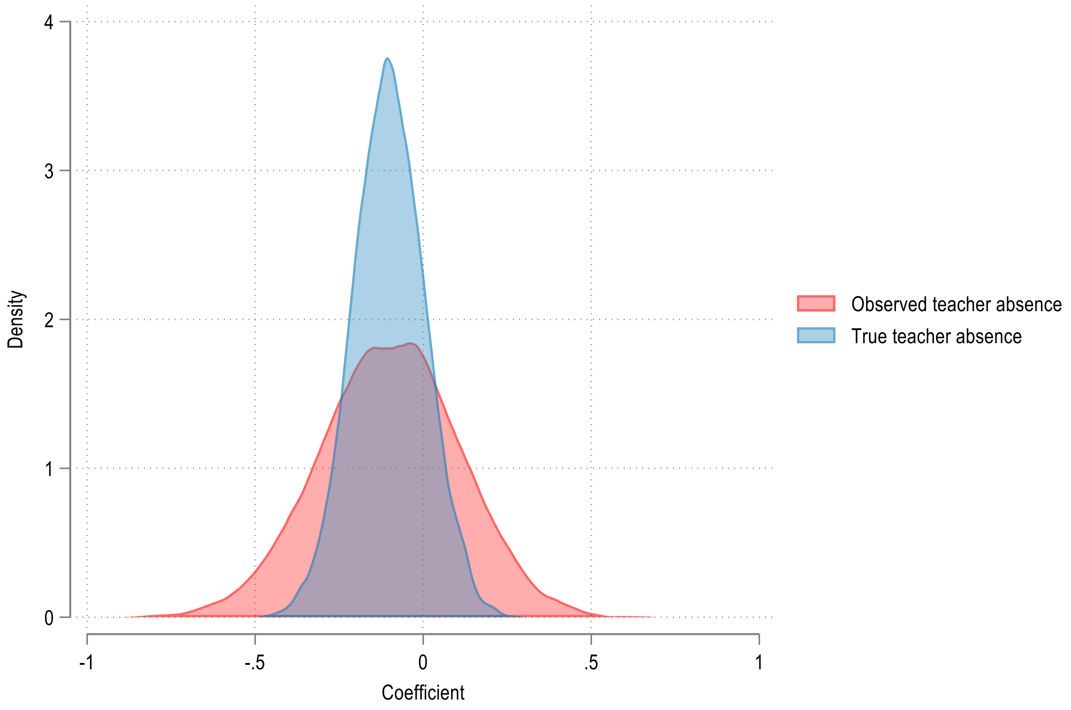

Appendix Figure C1: Distribution of the coefficients across the 10,000 draws.

Stata syntax to replicate Appendix Table C1 and Appendix Figure C1

```
clear all
version 17
set seed 89526

* DGP *
program define dgp
	syntax, schools(integer)
	qui {
		clear
		set obs `schools'
		gen school=_n
		gen u_school=rnormal(0,1)
		expand 3
		bysort school: gen grade=_n+7
		expand 10
		bysort school grade: gen time=_n
		gen t=u_school*-.01+rbeta(.9,20)+.05
		bysort school time: egen ts=mean(t)
		gen cohort=time if grade==8
		replace cohort=time-1 if grade==9
		replace cohort=time-2 if grade==10
		keep if inrange(cohort,1,8)
		bysort school cohort: egen observed=mean(ts)
		bysort school cohort: egen true=mean(t)
		cap frame drop temp
		frame create temp
		frame change temp
			clear
			set obs `schools'
			gen school=_n
			expand 8
			bysort school: gen cohort=_n
			expand 20
			gen id=_n
			expand 3
			bysort id: gen grade=_n +7
			tempfile id
			save `id'
		frame change default
		merge 1:m school cohort grade using `id', nogen
		keep if grade==10
		gen y=true*-.1+u_school+rnormal()
	}
end


* Data simulation *
frame create b str30 variable b
qui forvalues i=1/10000 {
	if `i'==1 nois _dots 0, title("Simulation scenario ") reps(`reps')
	nois _dots `i' 0
	dgp, schools(1000)
	areg y true, absorb(school)
	frame post b ("True teacher absence") (_b[true])
	areg y observed, absorb(school)
	frame post b ("Observed teacher absence") (_b[observed])
}

* Appendix Table C1 *
frame copy b table, replace
frame table {
	collapse (mean) b=b (sd) sd=b, by(variable)
	list
	putdocx begin, font(,10)
	putdocx paragraph
	putdocx text ("Table C1: "), bold
	putdocx text ("Average coefficient and standard deviation across 10,000 draws")
	putdocx table tbl1=data(variable b sd), varnames border(all,nil)			///
		layout(autofitcontents)
	putdocx table tbl1(1,1)=("")
	putdocx table tbl1(1,2)=("Coefficient")
```

```
        putdocx table tbl1(1,3)=("Standard deviation")
        putdocx table tbl1(1/1,.), bold border(top) border(bottom)
        putdocx table tbl1(.,2/3), nformat(%9.4f)
        putdocx table tbl1(3,.), border(bottom)
        putdocx save teachabs_sim_table_C1, replace
}


* Appendix Figure C1 *
frame b {
        tw      (kdensity b if variable=="Observed teacher absence",                           ///
                recast(area)  color(red%40))                                                   ///
                (kdensity b if variable=="True teacher absence",                               ///
                recast(area)  color(sea%40))                                                   ///
                , legend(order(1 " Observed teacher absence"                                   ///
                2 " True teacher absence" ))                                                   ///
                xtitle(Coefficient) ytitle(Density)
        graph export teachabs_sim_figure_C1.emf, replace
}
```

Online Appendix D: Bias by short-term teacher absence spells

This appendix simulates data to show that when only observing long-term absences, correlations between short- and long-term absences would likely lead to slightly upwardly biased estimates. As an illustration, consider a school with observed long-term absence rates of 3 and 5 percent for cohorts *A* and *B*. Further, suppose that unobserved short-term absences positively correlate with long-term absences: the cohorts with many long-term absences also have a lot of short-term absences. In that case, the actual difference in absence rate between the school cohorts *A* and *B* is larger than the observed two percentage points, and we overestimate the effects of the two-percentage-point increase in absence rate. (The opposite is the case if short- and long-term absences are negatively correlated, namely an underestimation.) In the following, we provide a simple simulation exercise to indicate bias caused by only observing long-term absences. Stata code to replicate the simulation results is included at the end of this appendix.

We construct 1,000 lower secondary schools s, each with 5 school cohorts c of 20 individuals i. From a subset of our data, we can observe all teacher absences, which allows us to get an estimate of the proportion of all absences that are short- (less than 16 days) and long-term (16 days or longer), as well as the within-school correlation between short- and long-term absences. Using this information, we draw samples from a multivariate normal distribution with the means of short- and long-term absences being 0.014 and 0.047 and their respective standard deviations being 0.005 and 0.01. From our data, we observe a within-school correlation of .2123 between short- and long-term absences; thus, we let short- and long-term absences be correlated .18. Compactly, we define the mean matrix $\boldsymbol{M}$ as $[.014 \quad .047]$ and the covariance matrix $\boldsymbol{V}$ as $\begin{bmatrix} .005^2 & .2123 * .005 * .01 \\ .2123 * .005 * .01 & .01^2 \end{bmatrix}$, and draw a random sample from a normal distribution $N(\boldsymbol{M}, \boldsymbol{V})$. Then, we replace negative values of short- and long-term absences with 0. Finally, we define the outcome as $y_{isc} = TL_{sc} * -.50 + TS_{sc} * -.50 + \varepsilon_{isc}$, where

$\varepsilon_{isc} \sim N(0,1)$ and where we assume that short- and long-term absences have the same impact on student outcomes. The analysis data consists of one observation for each individual (N=100,000), clustered in 1,000 schools and ten school cohorts.

Using a school fixed effects model, we compare the true effects of long-term absence (i.e., -0.50) with the estimated effects of long-term absence (β_1) from the following model across 10,000 independent draws, where β_0 is the constant and α_s is the school fixed effects:

$$y_{isc} = \beta_0 + \beta_1 TL_{sc} + \alpha_s + \epsilon_{isc}$$

The result in Appendix Table D1 below suggests that the estimated effects of long-term teacher absence are overestimated by 11.1% because of the correlation between short- and long-term teacher absences ($-0.5556/0.5$).

Appendix Table D1: Average coefficient and standard deviation across 10,000 draws

	**Coefficient**	**Standard deviation**
Observed long-term teacher absence effect	-0.5556	0.3540

Stata syntax to replicate Appendix Table D1

```
clear all
version 17
set seed 712971

* DGP *
program define dgp
        syntax, schools(integer)
        quietly {
                clear
                set obs `schools'
                generate school=_n
                expand 5
                bysort school: generate cohort=_n
                matrix m = (0.014,0.047)
                matrix sd = (0.005,0.01)
                local rho = .2123
                matrix C = (1, `rho'\ `rho',1)
                drawnorm s l, means(m) sds(sd) corr(C)
                replace s = 0 if s< 0
                replace l = 0 if l < 0
                expand 20
                generate id=_n
                generate y = -.50*l -.50*s + rnormal(0,1)

        }
end

* Data simulation *
frame create sim true estimate
quietly forvalues i=1/10000 {
        dgp, schools(1000)
        noisily : display `i' " " _continue
        areg y l, absorb(school)
        local b=_b[l]
        frame post sim (-.5) (`b')
}

* Appendix Table D1 *
frame copy sim table, replace
frame table {
        collapse (mean) b=estimate (sd) sd=estimate,
        gen str30 variable="Long-term teacher absence"
        list
        putdocx begin, font(,10)
        putdocx paragraph
        putdocx text ("Table D1: "), bold
        putdocx text ("Average coefficient and standard deviation across 10,000 draws")
        putdocx table tbl1=data(variable b sd), varnames border(all,nil)                    ///
                layout(autofitcontents)
        putdocx table tbl1(1,1)=("")
        putdocx table tbl1(1,2)=("Coefficient")
        putdocx table tbl1(1,3)=("Standard deviation")
        putdocx table tbl1(1/1,.), bold border(top) border(bottom)
        putdocx table tbl1(.,2/3), nformat(%9.4f)
        putdocx table tbl1(2,.), border(bottom)
        putdocx save teachabs_sim_table_D1, replace
}
```

Online Appendix E: Replication files

Stata 16.0 is used throughout this paper, and this supplementary online appendix includes Stata do-files to replicate all the results presented in the main text and in the supplementary appendices A and B (Stata code to replicate Appendix C and D are included in these appendices). The Norwegian register data used in this paper is made available for research by Statistics Norway. Access to the data requires that one is affiliated with an approved research institution and has the legal basis, as described here: see https://www.ssb.no/en/data-til-forskning/utlan-av-data-til-forskere. The code to replicate the results is available on SocArXiv (DOI 10.17605/OSF.IO/29N74). Readers can access Appendix E by using the following link (unzip the AppendixE.zip folder and read the instructions in the README.txt file):

https://doi.org/10.17605/OSF.IO/29N74
